# Recombinant bacterial *β*-galactosidases for lactose bioconversion and galacto-oligosaccharide production: a comparative overview

**DOI:** 10.3389/fmicb.2026.1943869

**Published:** 2026-10-12

**Authors:** Anel Kostanova, Yuliya Shamsiyeva, Zhiger Akishev, Bekbolat Khassenov, Annelya Tursunbekova

**Affiliations:** 1 Laboratory for Genetics and Biochemistry of Microorganisms, National Center for Biotechnology, Astana, Kazakhstan; 2 Institute of Engineering and Food Technology, S. Seifullin Kazakh Agrotechnical Research University, Astana, Kazakhstan; 3 Department of General Biology and Genomics, Institute of Natural Sciences, L.N. Gumilyov Eurasian National University, Astana, Kazakhstan; 4 Corporate Development and Strategy Department, S. Seifullin Kazakh Agrotechnical Research University, Astana, Kazakhstan

**Keywords:** galacto-oligosaccharides, industrial biotechnology, lactose bioconversion, protein engineering, recombinant enzymes, *β*-galactosidase

## Abstract

The increasing demand for lactose-free dairy products, sustainable whey valorization and high-value prebiotic ingredients has intensified the need for efficient bacterial *β*-galactosidases capable of both lactose hydrolysis and galacto-oligosaccharide (GOS) synthesis. Although numerous bacterial *β*-galactosidases have been identified and recombinantly produced, information on their structural characteristics, catalytic properties, recombinant production efficiency and performance under application-relevant conditions remains scattered across individual studies, complicating their comparative evaluation for specific biotechnological applications. This review presents a comprehensive comparative overview of recombinant bacterial *β*-galactosidases with particular emphasis on their potential for lactose bioconversion and GOS production. Current knowledge on enzyme diversity, glycoside hydrolase families, structural organization, molecular determinants of catalysis, recombinant expression systems, purification strategies, hydrolytic activity and transglycosylation capacity is critically evaluated together with recent advances in protein engineering, computational enzyme design and commercially available *β*-galactosidase preparations. Comparative analysis of published studies indicates that catalytic efficiency alone is insufficient to assess the potential suitability of *β*-galactosidases for industrial applications. Enzyme performance is influenced by the combined effects of molecular architecture, catalytic properties, recombinant production efficiency, thermostability, product inhibition and transglycosylation capacity. However, direct comparisons remain limited by differences in experimental conditions and by the relatively small number of studies conducted in real dairy matrices or under industrially relevant process conditions. Accordingly, this review compares the major biochemical, molecular, recombinant production and process-related factors that should be considered when evaluating *β*-galactosidases for lactose hydrolysis, GOS production and large-scale bioprocessing. Particular attention is given to the current limitations in translating laboratory-scale enzyme characterization into industrial application, including limited validation in complex dairy matrices, regulatory considerations, scalable catalyst configurations and downstream processing requirements. Current limitations of commercial *β*-galactosidase preparations and future opportunities offered by protein engineering, artificial intelligence-assisted enzyme discovery and structure-guided design are also discussed. Overall, this review provides a comparative perspective on recombinant bacterial *β*-galactosidases and identifies the principal factors influencing their potential application in lactose bioconversion and GOS production, while highlighting the need for further validation under application-relevant and industrial-scale conditions.

## Highlights

- Recombinant bacterial *β*-galactosidases are comparatively evaluated for lactose bioconversion and GOS production.
- Structural, catalytic and recombinant production characteristics are examined in relation to application-relevant performance.
- Performance in dairy matrices and concentrated lactose systems is distinguished from laboratory-scale enzyme characterization.
- Production systems, downstream processing and regulatory considerations influence the translation toward industrial applications.
- Protein engineering and computational approaches offer opportunities to improve application-relevant enzyme properties.

## 1 Introduction

The continuous expansion of the global dairy industry has substantially increased the generation of lactose-rich by-products, particularly cheese whey and whey permeate, posing significant environmental and economic challenges (Buchanan et al., 2023). Cheese whey and whey permeate are lactose-rich dairy side streams that are already valorized through multiple established routes, including whey protein products, lactose recovery, permeate powders and feed applications. Nevertheless, depending on their composition, degree of fractionation and local processing context, lactose-containing dairy streams can also serve as feedstocks for enzymatic bioconversion into value-added products. Where suitable valorization or treatment routes are unavailable, lactose-rich dairy effluents may also contribute to a high organic load, providing an additional incentive for efficient bioconversion strategies (Pires et al., 2021; Selmi et al., 2025). Consequently, the concept of whey valorization has emerged as a sustainable strategy aimed at converting lactose-containing dairy side streams into value-added products, including functional carbohydrates, fermented ingredients, bio-based chemicals and biofuels (Pires et al., 2021; Buchanan et al., 2023). Within this context, enzymatic lactose bioconversion has become one of the key technological approaches supporting circular bioeconomy concepts by simultaneously reducing environmental impact and generating industrially relevant products (Orrego and Klotz-Ceberio, 2022; Niamah et al., 2023).

The industrial importance of lactose bioconversion is closely associated with the rapidly growing demand for lactose-free dairy products and functional food ingredients (Li et al., 2023; Niamah et al., 2023). Approximately 65% of the world’s adult population exhibits varying degrees of lactose malabsorption, increasing the demand for lactose-free dairy products and efficient lactose bioconversion technologies (Misselwitz et al., 2019). Enzymatic hydrolysis using *β*-galactosidases has become the preferred industrial approach for lactose removal because it operates under mild processing conditions while preserving the nutritional and sensory quality of dairy products (Kalathinathan et al., 2023). In addition to improving lactose digestibility, *β*-galactosidases enhance the sweetness of milk through the formation of glucose and galactose and contribute to improved technological properties during dairy processing (Li et al., 2023; Costa et al., 2024). Consequently, *β*-galactosidases have become key industrial biocatalysts supporting both lactose-free dairy manufacturing and the production of value-added functional ingredients (Zhou et al., 2024).

Beyond lactose hydrolysis, increasing attention has been directed toward the transglycosylation activity of *β*-galactosidases, which enables the synthesis of galacto-oligosaccharides (GOS), one of the most extensively investigated, commercially valuable and clinically relevant prebiotics (Liu P. et al., 2025). GOS selectively stimulate the growth and metabolic activity of beneficial gut microorganisms, particularly species of *Bifidobacterium* and *Lactobacillus*, thereby promoting intestinal homeostasis, modulating immune responses and enhancing mineral absorption (Mei et al., 2022; Ignatova et al., 2025). Owing to these physiological benefits, GOS have been widely incorporated into infant formulas, functional foods and dietary supplements, leading to a rapidly expanding global market for prebiotic ingredients (Ambrogi et al., 2023). More broadly, recent developments in enzyme-enabled production of functional oligosaccharides demonstrate that precise control of enzyme specificity, product yield and degree of polymerization is becoming increasingly important for the scalable manufacture of next-generation prebiotic ingredients (Kostanova et al., 2026). Consequently, the industrial relevance of *β*-galactosidases is increasingly determined by their ability to balance efficient lactose hydrolysis with high transglycosylation capacity, rather than by hydrolytic activity alone (Iqbal et al., 2023; Sandra et al., 2026).

As the industrial applications of *β*-galactosidases continue to expand, the performance requirements imposed on these enzymes have become increasingly stringent. Industrial *β*-galactosidases are expected to combine high catalytic efficiency toward lactose, elevated transglycosylation capacity, broad pH adaptability, high thermostability, resistance to product inhibition and cost-effective recombinant production (Otter et al., 2022; Iqbal et al., 2023). However, naturally occurring *β*-galactosidases exhibit remarkable diversity in their structural organization, catalytic properties and biochemical characteristics, resulting in substantial differences in industrial performance (Liu P. et al., 2025). Consequently, no currently available bacterial *β*-galactosidase simultaneously satisfies all industrial requirements, and the selection of an appropriate *β*-galactosidase remains highly dependent on the intended industrial application (Singh and Sambyal, 2023).

Over the past decade, a substantial number of bacterial *β*-galactosidases have been identified and characterized from diverse microbial sources, including mesophilic, thermophilic and extremophilic bacteria. Numerous studies have characterized bacterial *β*-galactosidases with respect to their catalytic properties, structural organization, recombinant production strategies and potential industrial applications (Zhou et al., 2024; Király et al., 2025). However, these data remain highly fragmented because individual studies typically focus on a limited number of enzymes and evaluate different biochemical parameters under non-standardized experimental conditions, making direct comparisons difficult (Kalathinathan et al., 2023; Zhou et al., 2024). Moreover, currently available reviews generally address only specific aspects of *β*-galactosidases, such as enzyme sources, catalytic mechanisms, recombinant production or GOS synthesis, without comparatively integrating molecular, catalytic, production-related and application-related characteristics (Mei et al., 2022; Király et al., 2025). A comparative evaluation that considers molecular characteristics, catalytic performance, recombinant production and application-related parameters together may therefore provide a broader perspective on the factors influencing the potential technological use of recombinant bacterial *β*-galactosidases.

Rather than proposing a definitive decision tool for industrial enzyme selection, this review aims to identify and critically discuss the principal molecular, biochemical, production-related and process-related factors that should be considered when evaluating recombinant bacterial *β*-galactosidases for different biotechnological applications. The available evidence and its current limitations are considered in relation to lactose hydrolysis, GOS production and large-scale bioprocessing, while areas requiring further application-relevant and industrial-scale validation are highlighted.

## 2 Diversity, classification and evolution of bacterial *β*-galactosidases

### 2.1 Taxonomic diversity of bacterial *β*-galactosidases

#### 2.1.1 Expansion of bacterial *β*-galactosidase diversity through genomics and metagenomics

Recent advances in whole-genome sequencing, metagenomics and functional genome annotation have substantially expanded the known diversity of bacterial *β*-galactosidases. Unlike early studies, which primarily relied on culture-dependent isolation from dairy-associated microorganisms, modern genome mining and metagenomic approaches have revealed *β*-galactosidases across phylogenetically diverse bacteria inhabiting a wide range of ecological niches, considerably increasing the repertoire of enzymes available for recombinant production and industrial biotechnology (Coughlan et al., 2015; Poltronieri, 2018; Eberhardt et al., 2020).

The rapid accumulation of bacterial genome sequences has enabled systematic identification of putative *β*-galactosidase genes based on conserved catalytic motifs and glycoside hydrolase family classification, while functional metagenomics has facilitated the discovery of enzymes from previously uncultivable microorganisms (Cheng et al., 2017). Importantly, recent studies indicate that enzyme performance cannot be predicted solely from bacterial taxonomy. Closely related species may produce *β*-galactosidases with markedly different catalytic efficiencies, thermostability and transglycosylation capacities, whereas phylogenetically distant microorganisms can exhibit similar biochemical characteristics (Yang et al., 2025; Wang et al., 2026). Consequently, *β*-galactosidase discovery has shifted from traditional microorganism screening toward integrated genome-guided evaluation of sequence, structure and function, facilitating the selection of recombinant enzymes for specific industrial applications.

#### 2.1.2 β-Galactosidases from dairy-associated lactic acid bacteria

Among bacterial *β*-galactosidases, enzymes derived from lactic acid bacteria (LAB) remain the most extensively investigated because of their long history of safe use in food fermentation, generally recognized as safe (GRAS) status and natural adaptation to lactose-rich dairy environments. The established use of Lactobacillus species as starter cultures for controlling acidification, microbial safety and product quality further demonstrates their technological relevance in food biotechnology (Baytukenova et al., 2025). Many LAB species also have recognized food-grade or safety status, supporting their consideration as sources and potential production platforms for industrial enzymes (Ayub et al., 2026). Species belonging to the genera *Lactobacillus*, *Lacticaseibacillus*, *Lactiplantibacillus*, *Lactococcus* and *Streptococcus* produce *β*-galactosidases that efficiently hydrolyze lactose under conditions commonly employed in dairy processing, making LAB the principal source of food-grade enzymes for lactose hydrolysis and recombinant enzyme development (Yang et al., 2025).

Adaptation to milk has driven the evolution of *β*-galactosidases with high affinity toward lactose and optimal activity at near-neutral pH and moderate temperatures, characteristics well suited to the manufacture of lactose-free milk, fermented dairy products and whey-derived ingredients (Pagar et al., 2021; Czarniecka-Skubina et al., 2025). Several LAB enzymes also exhibit transglycosylation activity, enabling simultaneous lactose hydrolysis and galacto-oligosaccharide (GOS) synthesis under optimized reaction conditions (Zhao et al., 2025b).

Despite their common ecological origin, LAB-derived *β*-galactosidases display considerable diversity in catalytic efficiency, thermostability, substrate specificity and susceptibility to product inhibition (Tang et al., 2026; Wang et al., 2026). Consequently, bacterial taxonomy alone is insufficient to predict industrial performance, emphasizing the need for integrated evaluation of enzyme structure, catalytic properties and recombinant production characteristics when selecting *β*-galactosidases for specific biotechnological applications (Carro et al., 2021).

#### 2.1.3 *β*-galactosidases from intestinal bacteria

Intestinal microorganisms constitute another important source of bacterial *β*-galactosidases owing to their adaptation to the glycan-rich environment of the gastrointestinal tract. Species belonging to the genera *Bifidobacterium*, *Bacteroides*, *Akkermansia* and *Enterococcus* produce *β*-galactosidases that participate in the metabolism of lactose, human milk oligosaccharides (HMOs) and other dietary carbohydrates, thereby contributing to microbial colonization and ecological fitness within the gut (Dinleyici et al., 2023; Yan et al., 2024).

Among intestinal bacteria, *Bifidobacterium* species have been investigated most extensively because many strains encode multiple *β*-galactosidases with distinct substrate preferences and catalytic properties (Yan et al., 2024). This enzymatic diversity enables efficient utilization of structurally different oligosaccharides and has attracted considerable interest for the enzymatic production of galacto-oligosaccharides (GOS) and other functional carbohydrates. Consequently, *β*-galactosidases derived from gut microbiota are increasingly explored as promising biocatalysts for prebiotic synthesis and functional food applications (Ignatova et al., 2025; Zhao et al., 2025a). Compared with *β*-galactosidases from dairy-associated bacteria, intestinal enzymes generally exhibit broader substrate specificity reflecting adaptation to the chemically diverse carbohydrate environment of the gastrointestinal tract (Kazemi et al., 2025). This ecological specialization has expanded the diversity of recombinant *β*-galactosidases available for biotechnology and highlighted the gastrointestinal microbiota as an important reservoir of enzymes with potential applications beyond conventional lactose hydrolysis. Therefore, intestinal bacteria represent valuable sources of novel *β*-galactosidases for the development of next-generation recombinant biocatalysts (Movahedpour et al., 2022; Zhou et al., 2024).

#### 2.1.4 *β*-galactosidases from environmental and extremophilic bacteria

Environmental and extremophilic microorganisms have emerged as an important source of bacterial *β*-galactosidases with unique biochemical characteristics that are often unattainable in enzymes derived from dairy-associated bacteria. Species isolated from soil, marine environments, hot springs, geothermal habitats and polar ecosystems have evolved *β*-galactosidases adapted to extreme temperatures, pH values, salinity and other environmental stresses, considerably expanding the diversity of enzymes available for industrial biotechnology (Kumar A. et al., 2024; Guzmán et al., 2026).

Among these microorganisms, thermophilic species belonging to the genera *Geobacillus*, *Thermus* and *Bacillus* have received particular attention because their *β*-galactosidases exhibit high thermostability and prolonged catalytic activity at elevated temperatures (Ashaolu et al., 2025). Such properties are advantageous for industrial lactose hydrolysis, as high-temperature processes improve lactose solubility, reduce microbial contamination and enhance process efficiency. In contrast, psychrophilic bacteria produce cold-active *β*-galactosidases capable of maintaining high catalytic activity at refrigeration temperatures, making them attractive for the production of lactose-free refrigerated dairy products (Kalathinathan et al., 2023; Singh and Sambyal, 2023).

The remarkable diversity of environmental *β*-galactosidases demonstrates that ecological adaptation plays a central role in shaping enzyme functionality. Consequently, environmental and extremophilic bacteria have become valuable reservoirs of recombinant enzymes with desirable properties, including enhanced thermostability, cold activity and tolerance to harsh processing conditions. These characteristics complement those of dairy-associated *β*-galactosidases and considerably broaden the opportunities for selecting enzymes tailored to specific industrial applications (Mejía-Caballero et al., 2025).

#### 2.1.5 Summary

Collectively, bacterial *β*-galactosidases originate from taxonomically and ecologically diverse microorganisms, but ecological origin alone does not reliably predict industrial performance. Dairy-associated bacteria provide many enzymes adapted to lactose-rich matrices, intestinal bacteria expand the repertoire of enzymes with diverse substrate preferences and transglycosylation potential, whereas extremophilic microorganisms provide candidates with enhanced stability under demanding process conditions. Therefore, taxonomy and ecological origin should be regarded primarily as indicators for enzyme discovery rather than as stand-alone selection criteria. Representative examples are summarized in Table 1, while subsequent sections focus on the structural, catalytic and process-related characteristics that more directly determine industrial suitability.

**Table 1:** Selected characterized bacterial *β*-galactosidases representing ecological and functional diversity relevant to lactose bioconversion.

Bacterial genus	Representative species/strain	Ecological niche	GH family	Representative characteristic	Technological relevance	References
*Lactobacillus*	*Lactobacillus helveticus* *DSM 20075*	Dairy	GH2	Heterodimeric LacLM *β*-galactosidase; recombinant expression demonstrated in *E. coli* and *L. plantarum*; high transgalactosylation activity reported	High transglycosylation activity; recombinant production in *E. coli* and *L. plantarum*	Kittibunchakul et al. (2019)
*Lactococcus*	*L.lactis IL1403*	Dairy	GH2	Cold-active and acid-tolerant *β*-galactosidase; recombinant enzyme displayed high catalytic efficiency toward lactose and up to 98% lactose conversion	Low-temperature lactose hydrolysis in dairy processing	Vincent et al. (2013)
*Streptococcus*	*Streptococcus thermophilus* SDMCC050237	Dairy starter culture	GH2	BgaQ *β*-galactosidase; BgaQ-8012 mutation increased GOS synthesis from 20.5 to 26.7% and improved GOS formation in yogurt	GOS-enriched fermented dairy products	Zhao et al. (2022)
*Leuconostoc*	*L. mesenteroides*	Dairy/fermented foods	GH2	Dairy-associated *Leuconostoc* strains encode LacZ and/or LacLM *β*-galactosidases involved in lactose utilization	Lactose metabolism in dairy-associated LAB; source of additional *β*-galactosidase diversity	Schwab et al. (2010)
*Bifidobacterium*	*B. bifidum* S17	Intestinal tract	GH42	Trimeric BbgII β1 → 6-galactosidase; catalytic residues and lactose kinetics structurally characterized	Functional carbohydrate metabolism and candidate enzymes for GOS-related applications	Godoy et al. (2016)
*Bacillus*	*B. coagulans NL01*	Soil	GH42	Recombinant thermostable *β*-galactosidase with excellent lactose-hydrolysis capacity and demonstrated activity in whey	Elevated-temperature lactose hydrolysis and whey processing	Liu et al. (2019)
*Geobacillus*	*G. stearothermophilus*	Thermophilic habitats	GH42	Thermostable BgaB extensively studied by site-directed and saturation mutagenesis to modulate hydrolysis and transglycosylation	High-temperature lactose conversion and protein engineering	Dong et al. (2011)
*Thermus*	*T. thermophilus* A4	Hot-spring/thermophilic habitat	GH42	Thermostable homotrimeric *β*-galactosidase with experimentally resolved crystal structure and conserved catalytic residues	High-temperature biocatalysis and structural/mechanistic studies	Hidaka et al. (2002)
*Escherichia*	*Escherichia coli*	Intestinal/model bacterium	GH2	Classical LacZ *β*-galactosidase; extensively characterized structural and mechanistic model	Reference enzyme for mechanistic studies, recombinant expression and protein engineering	Juers et al. (2001)
*Pantoea*	*P. anthophila*	Plant-associated/fermented beverage isolate	GH42	Recombinant *β*-galactosidase synthesized β(1 → 3)- and β(1 → 6)-linked GOS from both lactose and whey	GOS production from lactose-rich and whey-derived substrates	Yañez-Ñeco et al. (2021)

The enzymes included in table were selected as representative examples of bacterial β-galactosidases from different ecological and taxonomic groups and are not intended to constitute a comprehensive taxonomic survey. Reported functional characteristics derive from study-specific experimental conditions and should therefore be interpreted qualitatively rather than as directly comparable quantitative performance metrics. GH-family assignments were verified against the cited primary studies and/or the CAZy database.

Table 1 illustrates the broad ecological and functional diversity of characterized bacterial *β*-galactosidases. However, because the reported biochemical properties were obtained under heterogeneous experimental conditions, these examples should be interpreted qualitatively rather than as a direct quantitative ranking of enzyme performance. The following section therefore shifts from taxonomic origin to GH-family classification and structural organization.

### 2.2 Glycoside hydrolase families of bacterial *β*-galactosidases

#### 2.2.1 Overview of glycoside hydrolase classification

Bacterial *β*-galactosidases are classified in the Carbohydrate-Active enZYmes (CAZy) database according to amino acid sequence similarity, conserved catalytic residues and structural organization rather than substrate specificity alone (Masasa et al., 2022). Consequently, enzymes catalyzing the same reaction may belong to different glycoside hydrolase (GH) families.

To date, bacterial *β*-galactosidases have been identified mainly within four GH families: GH1, GH2, GH35 and GH42. Although all hydrolyze *β*-galactosidic bonds, they differ in structural organization, substrate specificity, thermostability and industrial relevance (Gotoh et al., 2023). Among them, GH2 and GH42 comprise most industrially relevant enzymes, whereas GH1 and GH35 contribute additional structural and functional diversity. An overview of the classification and distinguishing characteristics of these families is presented in Figure 1.

**Figure 1:**
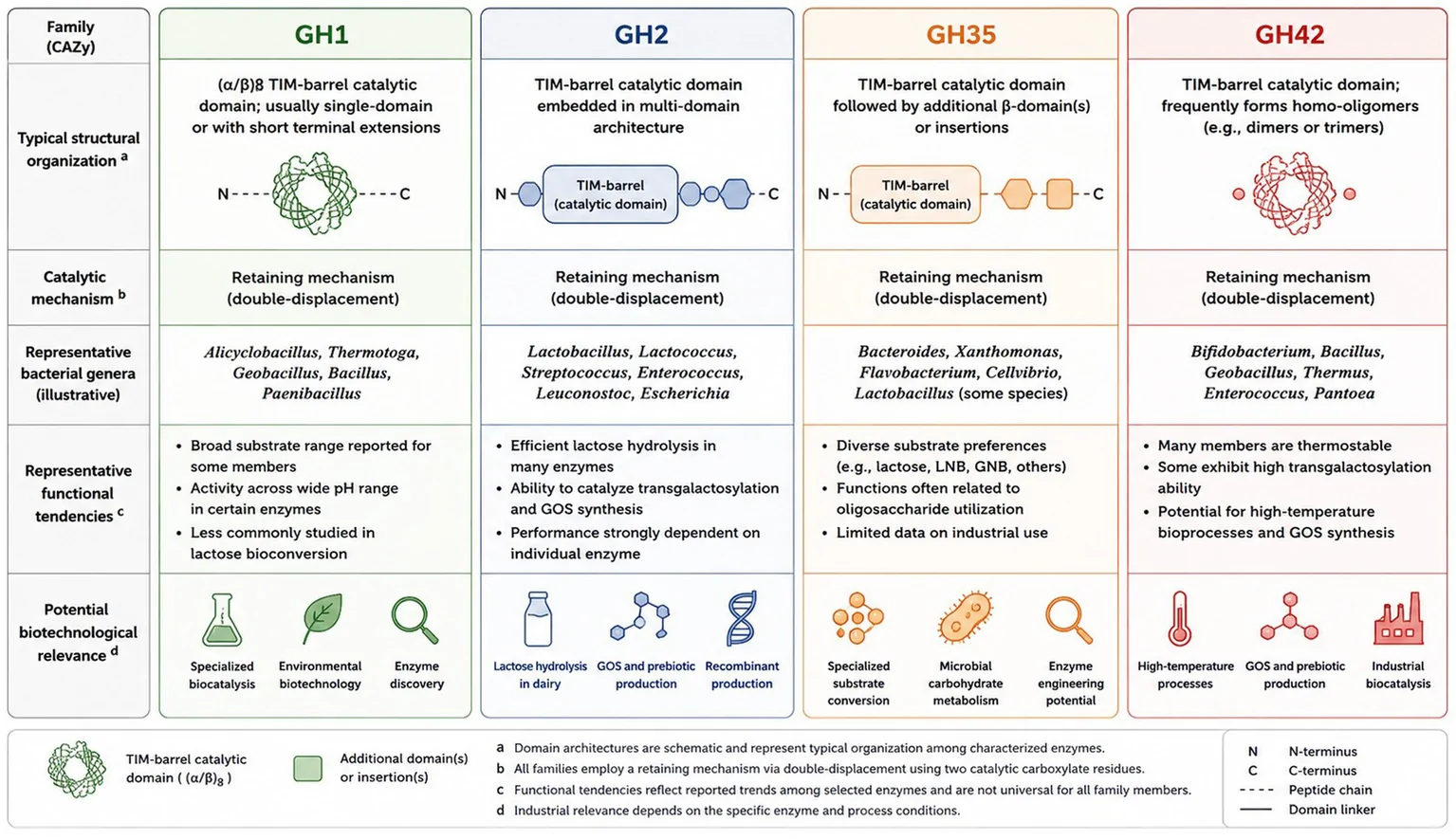
Classification and comparative characteristics of bacterial *β*-galactosidases belonging to glycoside hydrolase families GH1, GH2, GH35 and GH42.

Figure 1 summarizes the principal structural and functional characteristics of the four major glycoside hydrolase families of bacterial *β*-galactosidases. Although all families catalyze the same reaction, they differ considerably in molecular architecture and industrial relevance.

#### 2.2.2 GH1 family

GH1 represents an evolutionarily conserved glycoside hydrolase family that includes comparatively few bacterial *β*-galactosidases (Polaina and Talens-Perales, 2026). Bacterial GH1 enzymes have mainly been identified in thermophilic and environmental microorganisms and are characterized by a conserved (β/α)₈ TIM-barrel fold with two catalytic glutamate residues (Liu et al., 2026). Although less studied than GH2 and GH42, GH1 *β*-galactosidases expand the structural diversity of bacterial enzymes and represent promising targets for future protein engineering and specialized biotechnological applications (Shafi and Husain, 2023).

#### 2.2.3 GH2 family

The GH2 family comprises the largest and most extensively characterized group of bacterial *β*-galactosidases, including most enzymes used for industrial lactose hydrolysis and galacto-oligosaccharide (GOS) production (Liu et al., 2024; Zhou et al., 2024). GH2 enzymes are characterized by a multidomain architecture and generally exhibit high catalytic efficiency toward lactose together with considerable transglycosylation activity. Owing to their favorable biochemical properties and extensive characterization, GH2 *β*-galactosidases represent the principal targets for recombinant production and industrial dairy applications (Wang et al., 2022).

#### 2.2.4 GH35 family

Compared with other glycoside hydrolase families, GH35 contains relatively few bacterial *β*-galactosidases and has been investigated less extensively (Saburi et al., 2023). These enzymes are mainly identified in environmental and intestinal bacteria, where they contribute to carbohydrate metabolism and glycan degradation. Although GH35 *β*-galactosidases have limited industrial application at present, their distinct structural organization and catalytic properties broaden the functional diversity of bacterial *β*-galactosidases and provide additional opportunities for enzyme discovery and engineering (Liu et al., 2024; Zhou et al., 2024).

#### 2.2.5 GH42 family

The GH42 family includes numerous bacterial *β*-galactosidases derived from thermophilic microorganisms and *Bifidobacterium* species, many of which exhibit high thermostability and efficient transglycosylation activity (Moura et al., 2007; Kittibunchakul et al., 2020; Yan et al., 2024). These characteristics make GH42 enzymes particularly attractive for high-temperature lactose hydrolysis and GOS production. Consequently, GH42 *β*-galactosidases have become important targets for recombinant production and industrial biotechnology, complementing the widespread application of GH2 enzymes (Duan et al., 2023).

#### 2.2.6 Comparative analysis of GH families

Although GH1, GH2, GH35 and GH42 *β*-galactosidases catalyze the same reaction, they differ markedly in structural organization, catalytic properties and industrial relevance. GH2 and GH42 represent the principal bacterial families used in lactose hydrolysis and galacto-oligosaccharide (GOS) production owing to their high catalytic efficiency, thermostability and transglycosylation capacity. In contrast, GH1 and GH35 are less abundant and remain comparatively underexplored but contribute.

additional structural diversity and potential for enzyme engineering (Kalathinathan et al., 2023; Liu et al., 2024; Zhou et al., 2024). Therefore, GH family classification provides an important basis for enzyme selection; however, structural and biochemical characterization remains essential for identifying the most suitable recombinant *β*-galactosidase for specific industrial applications.

### 2.3 Evolutionary relationships and phylogenetic diversity

#### 2.3.1 Evolutionary diversification

Bacterial *β*-galactosidases exhibit substantial evolutionary diversity, as reflected by their distribution across multiple glycoside hydrolase families and by considerable sequence and structural variation among enzymes catalyzing *β*-galactoside hydrolysis. GH-family classification therefore represents evolutionary and structural relationships among enzymes rather than a simple classification based on substrate specificity (Lombard et al., 2014; Zhou et al., 2024). Within individual GH families, sequence diversification, changes in domain organization and variation in substrate-recognition regions have generated enzymes with distinct biochemical properties. Consequently, evolutionary relatedness may provide useful information for identifying homologous enzymes and conserved structural features, but it should not be used independently to predict catalytic or industrial performance.

#### 2.3.2 Conservation of catalytic machinery and functional divergence

Despite extensive sequence divergence, retaining *β*-galactosidases preserve the general catalytic requirement for two carboxylate residues, typically glutamates, acting as the catalytic nucleophile and general acid/base catalyst. However, the positions and surrounding sequence environments of these catalytic residues differ among GH families and individual enzymes; therefore, residue numbering cannot be generalized across bacterial *β*-galactosidases (Lombard et al., 2014; Kalathinathan et al., 2023). In the extensively characterized GH2 *Escherichia coli* LacZ *β*-galactosidase, for example, Glu537 functions as the catalytic nucleophile and Glu461 as the acid/base catalyst (Juers et al., 2001). Variations in residues surrounding the catalytic centre, substrate-binding regions and additional structural domains can modify substrate recognition and catalytic behaviour without altering the fundamental retaining mechanism. Thus, conservation of catalytic machinery can coexist with substantial functional diversification among *β*-galactosidases.

#### 2.3.3 Evolutionary information in application-oriented enzyme selection

Evolutionary and sequence relationships can support recombinant *β*-galactosidase discovery by identifying homologous enzymes, conserved catalytic features and sequence-divergent candidates for subsequent biochemical characterization. However, phylogenetic proximity should not be interpreted as evidence of equivalent industrial performance. Even homologous *β*-galactosidases may differ in catalytic efficiency, substrate preference, temperature and pH dependence, product inhibition and transglycosylation behaviour. Therefore, phylogenetic analysis is most informative when integrated with structural, kinetic and process-related data rather than used as an independent predictor of industrial performance. In the present framework, phylogenetic analysis is consequently used to contextualize sequence diversity among representative GH2 LacZ proteins, whereas their technological suitability must be evaluated using experimentally determined biochemical and process parameters (Lombard et al., 2014; Király et al., 2025).

#### 2.3.4 Phylogenetic context of representative GH2 LacZ *β*-galactosidases

To provide evolutionary context for the sequence diversity discussed above, a phylogenetic analysis was performed using representative full-length GH2 LacZ *β*-galactosidase sequences (Supplementary Figure S1). The resulting tree illustrates sequence-level relationships among the selected enzymes. However, because phylogenetic proximity alone does not reliably predict catalytic efficiency, transglycosylation capacity, stability or process performance, the analysis is used here as evolutionary context rather than as an independent predictor of industrial performance (Kumar S. et al., 2024).

The phylogenetic analysis revealed several sequence-level groupings among the selected GH2 LacZ *β*-galactosidases (Supplementary Figure S1). Several enzymes from closely related taxa clustered together, including representatives of *Lactobacillus*, *Lactococcus* and *Bifidobacterium*, whereas some sequences from taxonomically distinct bacteria occurred within broader shared lineages. Because the analysis was based on a single protein family, these relationships should be interpreted as patterns of similarity among the selected GH2 LacZ sequences rather than as a reconstruction of bacterial species phylogeny (Lombard et al., 2014; Zhou et al., 2024). Importantly, phylogenetic proximity alone does not demonstrate similarity in lactose-hydrolysis efficiency, transglycosylation capacity, thermostability or other technologically relevant properties (Kalathinathan et al., 2023; Zhou et al., 2024). Accordingly, the phylogenetic analysis is used here to provide evolutionary context for GH2 sequence diversity, whereas industrial applicability should be assessed using experimentally determined structural, catalytic and process-related characteristics discussed in the subsequent sections.

## 3 Structural and molecular determinants governing *β*-galactosidase performance

### 3.1 Catalytic residues and reaction mechanism

The structural organization of bacterial *β*-galactosidases is a major determinant of substrate recognition, catalytic behaviour, stability and transglycosylation potential. Although *β*-galactosidases from different glycoside hydrolase families catalyse related *β*-galactosidic bond-cleavage reactions, their overall architectures differ substantially in domain composition, oligomeric state and active-site organization (Lombard et al., 2014; Kalathinathan et al., 2023).

Members of GH1, GH2, GH35 and GH42 share a retaining catalytic mechanism and contain a catalytic (β/α)₈ TIM-barrel domain, but the arrangement and number of accessory domains vary considerably among families and individual enzymes. In GH35, for example, the catalytic TIM-barrel may be followed by one or more *β*-rich auxiliary domains rather than being replaced by a *β*-sandwich catalytic fold. Structurally characterized GH35 enzymes from *Lacticaseibacillus casei* and *Streptococcus pneumoniae* illustrate this modular organization and its relationship to substrate recognition (Saburi et al., 2023).

GH2 *β*-galactosidases are frequently multidomain enzymes. A representative example is BgaD from *Bacillus circulans* ATCC 31382, a retaining GH2 *β*-galactosidase with a large multidomain architecture. Structural analysis showed a TIM-barrel catalytic domain together with additional *β*-sheet-rich domains, while mutational studies identified Glu447 as the general acid/base catalyst and Glu532 as the catalytic nucleophile. These structural and active-site features are directly relevant to substrate positioning, catalytic activity and transgalactosylation behaviour (Bultema et al., 2014).

Oligomeric assembly provides an additional level of structural organization. For example, Gan42B from *Geobacillus stearothermophilus* is a homotrimeric GH42 *β*-galactosidase whose active sites are formed at interfaces between neighbouring subunits. Its crystal structure demonstrates how oligomerization contributes directly to active-site architecture and may influence enzyme stability and function under elevated-temperature conditions (Dong et al., 2011; Kalathinathan et al., 2023). Thermophilic GH42 *β*-galactosidases from *Geobacillus* species provide representative examples in which extensive subunit interactions contribute substantially to thermal resistance while preserving catalytic activity at elevated temperatures (Solomon et al., 2015).

Collectively, these examples demonstrate that *β*-galactosidase performance cannot be inferred from GH-family assignment alone. Domain organization, active-site geometry, oligomeric assembly and local residue interactions must be considered together with experimentally determined catalytic and process-related parameters. These relationships are summarized schematically in Figure 2.

**Figure 2:**
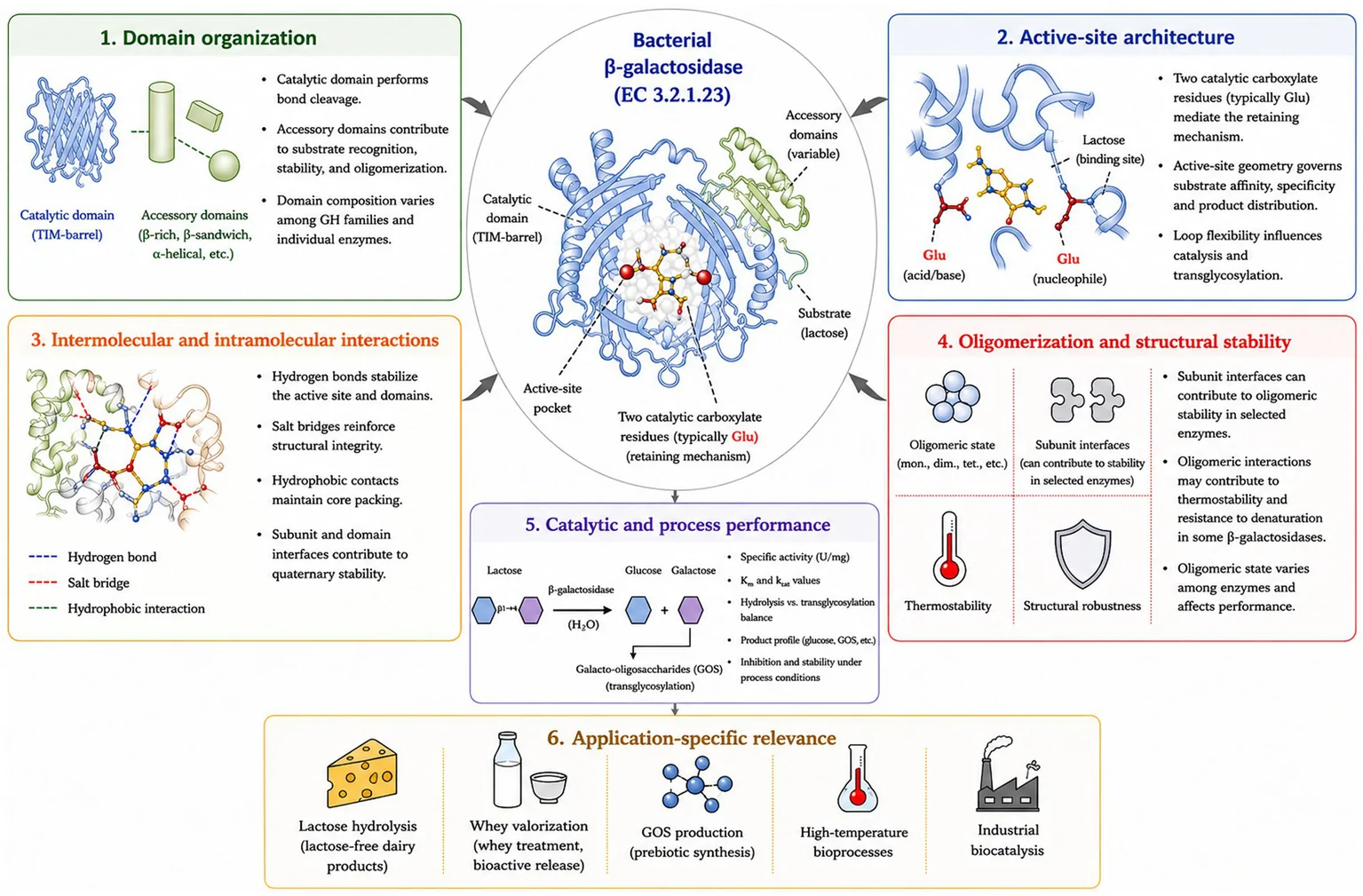
Conceptual representation of structural and molecular determinants influencing bacterial *β*-galactosidase performance.

Figure 2 illustrates general relationships among domain organization, active-site architecture, molecular interactions, oligomeric assembly, catalytic behaviour and application-specific performance. It is a conceptual synthesis based on structurally and biochemically characterized *β*-galactosidases and is not derived from a single experimental protein structure. Representative structural relationships are supported by studies of GH2 BgaD from *B. circulans*, GH35 *β*-galactosidases, and GH42 Gan42B from *G. stearothermophilus* (Bultema et al., 2014; Solomon et al., 2015; Yin et al., 2017; Saburi et al., 2023).

### 3.2 Catalytic mechanism and hydrolysis–transgalactosylation partitioning

#### 3.2.1 Catalytic residues and retaining mechanism

Despite substantial sequence and structural diversity, bacterial *β*-galactosidases belonging to retaining GH families use two catalytic carboxylate residues, most commonly glutamates, as the catalytic nucleophile and general acid/base catalyst. Their exact residue positions differ among individual enzymes and GH families. In the GH2 *β*-galactosidase BgaD from *B. circulans* ATCC 31382, mutational analysis identified Glu532 as the catalytic nucleophile and Glu447 as the general acid/base catalyst (Bultema et al., 2014). During the first displacement step, the nucleophile attacks the anomeric centre of the galactosyl moiety, generating a covalent galactosyl–enzyme intermediate; during the second displacement step, this intermediate is resolved by an incoming acceptor, while the overall anomeric configuration is retained.

Catalytic performance is determined not only by these catalytic residues but also by surrounding active-site residues involved in substrate binding, positioning and product specificity. In *B. circulans* BgaD-D, site-directed mutagenesis demonstrated that residues surrounding the catalytic centre influence substrate positioning and GOS linkage specificity, providing direct experimental evidence that the broader active-site environment contributes substantially to transgalactosylation behaviour (Yin et al., 2017).

#### 3.2.2 Competing hydrolysis and transgalactosylation pathways

*β*-galactosidases catalyse hydrolysis and transgalactosylation through the same covalent galactosyl–enzyme intermediate. In hydrolysis, water acts as the acceptor during degalactosylation, whereas in transgalactosylation the galactosyl group is transferred to lactose or another carbohydrate acceptor, generating GOS or related products. The relative contribution of these pathways is strongly dependent on enzyme-specific active-site properties and reaction conditions.

Increasing lactose concentration generally favours transgalactosylation because carbohydrate acceptors compete more effectively with water for the galactosyl intermediate. This creates an important process distinction between direct lactose hydrolysis in milk, whey or dilute whey permeate and industrial GOS synthesis, which typically employs concentrated lactose feeds; GOS-production studies commonly use lactose concentrations above approximately 200 g/L, although the optimum is strongly enzyme- and process-dependent. The extent of this shift is additionally influenced by temperature, pH, enzyme loading, water activity and accumulating reaction products such as glucose, galactose and GOS (Warmerdam et al., 2014; Martins et al., 2019).

#### 3.2.3 Structural control of product specificity

Beyond substrate concentration, active-site architecture influences how efficiently carbohydrate acceptors compete with water and which glycosidic linkages are formed. Mutational analysis of *B. circulans* BgaD-D identified residues involved in substrate binding and positioning that alter enzyme activity and GOS linkage specificity (Yin et al., 2017).

These findings support structure-guided engineering of *β*-galactosidases for targeted GOS product profiles. However, relationships between active-site geometry, loop flexibility and hydrolysis, transgalactosylation partitioning should not be generalized across unrelated *β*-galactosidases without enzyme-specific experimental validation.

### 3.3 Structure–function relationships and process implications

The functional properties of bacterial *β*-galactosidases emerge from the combined effects of active-site architecture, domain organization, protein dynamics and oligomeric assembly. Consequently, enzymes sharing the same catalytic mechanism or GH-family assignment can differ substantially in lactose affinity, catalytic turnover, substrate specificity, thermostability and transgalactosylation behaviour.

Structural studies provide direct examples of these relationships. In GH2 BgaD from *B. circulans*, active-site residues surrounding the catalytic glutamates participate in substrate positioning and determine GOS linkage specificity (Bultema et al., 2014; Yin et al., 2017).

In GH35 LBCZ_0230 from *Lacticaseibacillus casei*, structural and biochemical characterization demonstrated a specialized substrate-recognition architecture associated with high activity toward lacto-N-biose I and galacto-N-biose rather than conventional lactose-processing behaviour (Saburi et al., 2023).

These examples also illustrate why generalized structural rules should be applied cautiously. Oligomerization, loop rigidity, salt bridges or hydrophobic packing may contribute to stability in particular enzymes, but they should not be treated as universal predictors of industrial performance across unrelated *β*-galactosidases. Structural information is most informative when linked to experimentally determined kinetic, stability and process data for the same enzyme.

From an application-oriented perspective, structural characteristics therefore provide mechanistic explanations for observed enzyme behaviour but should complement rather than replace quantitative performance measurements. Lactose-specific kinetic parameters, operational stability, product inhibition, GOS yield and product profile, together with performance in milk, whey or concentrated lactose feeds, remain necessary for assessing industrial suitability. The principal experimentally supported structure–function relationships are summarized in Table 2.

**Table 2:** Structural and catalytic determinants governing bacterial *β*-galactosidase performance.

Structural determinant	Molecular role	Influence on enzyme performance	Representative examples	References
Catalytic carboxylate residues	Nucleophile and general acid/base catalyst	Essential for catalytic turnover	*B. circulans* BgaD: Glu532 identified as nucleophile and Glu447 as acid/base catalyst	Bultema et al. (2014)
Active-site residues surrounding catalytic centre	Substrate positioning and acceptor orientation	Activity and GOS linkage specificity	*B. circulans* BgaD-D active-site mutants altered product profiles	Yin et al. (2017)
Catalytic-domain architecture	Formation of substrate-binding/catalytic pocket	Substrate-recognition geometry and specificity	GH35 LBCZ_0230 from *L. casei* preferentially hydrolyses LNB and GNB	Saburi et al. (2023)
Accessory domains	Contribute to substrate recognition and protein organization	Can modify substrate binding and product specificity	Multidomain GH2 BgaD from *B. circulans*	Bultema et al. (2014)
Oligomeric assembly	Formation/stabilization of quaternary structure	Can influence active-site organization and structural stability	Selected GH42 β-galactosidases	Solomon et al. (2015)
Local hydrogen-bonding/electrostatic interactions	Stabilization of substrate and catalytic intermediates	Catalytic efficiency and transition-state stabilization	Histidine and glutamate residues in *B. circulans* BgaD	Yin et al. (2017)

As summarized in Table 2, experimentally characterized *β*-galactosidases demonstrate that catalytic performance arises from the combined effects of catalytic residues, substrate-binding architecture, domain organization and oligomeric assembly. These structural determinants should not be interpreted as universal predictors of industrial performance across unrelated enzymes; their relevance is most informative when linked to enzyme-specific kinetic, stability and process data.

### 3.4 Catalytic properties

#### 3.4.1 Lactose-specific kinetic performance

Quantitative comparison of bacterial *β*-galactosidases is complicated by substantial heterogeneity in experimental design. Published studies differ in assay substrate, substrate concentration, temperature, pH, buffer composition, enzyme purity and the kinetic parameters reported. In particular, kinetic values obtained with chromogenic substrates such as o-nitrophenyl-*β*-D-galactopyranoside (oNPG) should not be interpreted as direct measures of catalytic performance toward lactose. Several *β*-galactosidases exhibit markedly different kinetic behaviour toward oNPG and lactose, demonstrating that substrate-specific measurements are essential when evaluating enzymes for dairy lactose bioconversion.

For example, the recombinant *β*-galactosidase from *Lactobacillus helveticus* DSM 20075 displayed substantially different kinetic parameters toward lactose and oNPG, illustrating that high activity toward a synthetic substrate does not necessarily translate into equivalent lactose-processing performance (Kalathinathan et al., 2023). Therefore, the quantitative comparison presented in Table 3 is restricted to kinetic parameters determined using lactose as the substrate. Where available, information on the assay matrix and direct validation in milk or whey is additionally provided to facilitate assessment of industrial relevance.

**Table 3:** Reported lactose-specific kinetic parameters of representative bacterial *β*-galactosidases.

Source organism	GH family	Recombinant expression host	Assay matrix/substrate	Assay conditions	*K_m_* (mM)	*V_max_*	*k_cat_* (_S_^-1^)	*k_cat_/K_m_* (_mM_^-1^_S_^-1^)	Application- and matrix-specific hydrolysis/transgalactosylation evidence	References
*Lactobacillus helveticus* DSM 20075	GH2	*Escherichia coli* BL21 Star (DE3)	Lactose, buffer	Kinetic assay conditions as reported by authors	15.7 ± 1.3	11.1 ± 0.2 μmol glucose min^-1^ mg^-1^	20.2 ± 0.3	1.29	GOS formation and dairy-processing relevance demonstrated	Kittibunchakul et al. (2019)
*Bifidobacterium breve* DSM 20213 *β*-gal I	GH2	*Escherichia coli* + GroEL/GroES	Lactose, buffer	50 mM sodium phosphate buffer, pH 6.5, 30 °C	15.3 ± 3.2	59 ± 2 μmol min^-1^ mg^-1^	114 ± 4	7.4 ± 1.9	33% total GOS yield reported	Arreola et al. (2014)
*B. breve* DSM 20213, *β*-gal II	GH2	*Escherichia coli* + GroEL/GroES	Lactose, buffer	50 mM sodium phosphate buffer, pH 6.5, 30 °C	7.5 ± 0.9	97 ± 5 μmol min^-1^ mg^-1^	188 ± 10	25 ± 4	44% total GOS yield reported	Arreola et al. (2014)
*Bifidobacterium breve* DSM 20213, Bbreβgal-III (WT)	GH42	*Escherichia coli*	Lactose, buffer	50–800 mM lactose; 50 mM NaPB, pH 6.5; 30 °C; 10 min	30 ± 3	NR	85 ± 5	2.8 ± 0.2	Limited transgalactosylation; maximum GOS yield 17% of total sugars for WT	Phirom-on et al. (2026)
*Streptococcus thermophilus* DSM 20259, LacZ	GH2	*Lactiplantibacillus plantarum* WCFS1	Lactose, buffer	50 mM sodium phosphate buffer, pH 6.5, 30 °C; lactose 0–600 mM	5.12 ± 0.53	17.67 ± 0.36 μmol glucose min^-1^ mg^-1^	34.4 ± 0.70	6.7	Whey permeate: maximum GOS yield ~50% of total sugar mass at ~90% lactose conversion from 205 g L^−1^ initial lactose at 50 °C	Geiger et al. (2016)
*Alteromonas* sp. ML52, Gal	GH2	*Escherichia coli* BL21(DE3)	Lactose, buffer	35°C; 50 mM sodium phosphate buffer, pH 8.0; lactose 0.2–40 mM	7.2	18.5 U mg^-1^	146.5	20.3	>90% lactose hydrolysis in milk after 24 h at 4 °C and 10 °C; 94% after 5 h at 25 °C	Sun et al. (2018)
*Paracoccus* sp. 32d, BgaL	GH2	*Escherichia coli* (pBAD expression system)	Lactose, buffer	10 °C; lactose 1.0–5.0 mM	2.94 ± 0.39	NR	43.23 ± 1.13	NR	~97% lactose hydrolysis in milk after 24 h at 10 °C using 1 U enzyme	Wierzbicka-Woś et al. (2011)
*Arthrobacter psychrolactophilus* F2, rBglAp	GH2	*Escherichia coli*	Lactose, buffer	10 °C	42.1	NR	NR	NR	~70% lactose hydrolysis in 1 mL milk after 24 h using 1 U enzyme; trisaccharide formation from lactose demonstrated	Nakagawa et al. (2007)
*Bacillus coagulans* MA-13, BcGalB	GH42	*Escherichia coli* Rosetta™ (DE3) pLysS, pET28b/BcGalB	D-lactose, buffer	100 mM sodium citrate, pH 5.0; 60 °C; lactose 0–500 mM	136.2	NR	1603.7	11.8	Specific activity toward lactose = 1,283 U mg^-1^; homo- and hetero-transglycosylation/GOS synthesis demonstrated	Aulitto et al. (2021)
*Paenibacillus wynnii*, BgaPw	NR	*Escherichia coli* BL21(DE3), pET20b_BgaPwHis6	Lactose; Novo buffer and milk diluted with Novo buffer	8 °C; lactose 0.76–97.5 mM for *K_m_* determination	0.34 ± 0.084 (buffer) / 0.63 ± 0.045 (milk)	NR	NR	NR	>99.7% lactose hydrolysis in milk after 72 h at 8 °C using 2.7 nkat mL^−1^; 144 g L^−1^ GOS from 400 g L^−1^ lactose after 24 h (36.0% yield)	Lutz-Wahl et al. (2024)

Among lactose-specific parameters, *K_m_*, *k*_*ca*t_, and *k_cat_/K_m_* provide complementary information on apparent substrate affinity, catalytic turnover and catalytic efficiency. However, even lactose-derived values should not be treated as strictly interchangeable between studies because they may have been determined under different temperatures, pH values, enzyme concentrations and kinetic models. Accordingly, Table 3 should be interpreted as a literature-based comparison of reported enzyme-specific kinetic behaviour rather than as an absolute ranking of *β*-galactosidase performance (Geiger et al., 2016).

#### 3.4.2 Comparability and limitations of reported kinetic parameters

Direct comparison of published kinetic parameters requires particular caution when different enzyme preparations, assay substrates and experimental conditions are used. *V*_*m*ax_ expressed per milligram of protein is dependent on the enzyme preparation and protein quantification, whereas *k_cat_* requires knowledge of the molar concentration of catalytically competent enzyme. Moreover, kinetic parameters obtained with synthetic chromogenic substrates such as o-nitrophenyl-*β*-D-galactopyranoside (oNPG) should not be directly extrapolated to lactose. For example, recombinant *β*-galactosidase from *Streptococcus thermophilus* showed a *V*_*m*ax_ of 17.67 μmol min^-1^ mg^-1^ and a *k_cat_*/*K_m_* of 6.7 mM^-1^ s^-1^ toward lactose, compared with 151 μmol min^-1^ mg^-1^ and 539 mM^-1^ s^-1^, respectively, toward oNPG, demonstrating the strong substrate dependence of apparent catalytic performance (Geiger et al., 2016).

Another important limitation is that kinetic constants determined in buffered model systems do not necessarily predict enzyme performance in industrial dairy matrices. Milk, whey and whey permeate differ in composition and physicochemical environment, and enzyme performance may therefore depend on the actual processing matrix as well as temperature, enzyme loading and reaction time. Experimental studies performed directly in milk, cheese whey and whey permeate have demonstrated matrix-dependent differences in lactose hydrolysis (Dutra Rosolen et al., 2015). More recent work with recombinant *β*-galactosidase from Marinomonas sp. ef1 demonstrated high lactose-hydrolysis efficiency in real dairy substrates, with reported hydrolysis yields of 82% in skimmed milk at 4 °C and 94% in cheese whey permeate at 50 °C (Bianchi et al., 2024). Consequently, kinetic characterization in buffer should be complemented by measurements of lactose conversion, operational stability and, where relevant, transgalactosylation/GOS formation in the intended dairy matrix.

#### 3.4.3 Industrial interpretation of kinetic performance

Lactose-specific catalytic efficiency is useful for identifying promising *β*-galactosidases, particularly under substrate concentrations below saturation, but it should not be used as an isolated predictor of industrial performance. Direct lactose hydrolysis and GOS synthesis impose fundamentally different process requirements. For lactose hydrolysis in milk or whey, catalytic activity must be considered together with operational temperature, enzyme stability, product inhibition and performance in the actual dairy matrix. Recent studies with recombinant bacterial *β*-galactosidases demonstrate that high lactose conversion can be achieved directly in technologically relevant matrices, including skimmed milk and cheese whey permeate (Bianchi et al., 2024).

In contrast, GOS synthesis generally requires substantially higher initial lactose concentrations to promote transgalactosylation relative to hydrolysis. A recent systematic review and meta-analysis integrating 104 studies published between 2018 and 2024 identified 30–50% (w/v) lactose as a favourable range for GOS biosynthesis, although the optimal conditions remain enzyme-specific (Costa et al., 2025). Consequently, kinetic parameters determined under low-substrate conditions cannot alone predict performance during high-solids GOS production, where substrate concentration, enzyme loading, temperature, pH, reaction time and product composition become critical process variables.

Accordingly, kinetic parameters should be interpreted together with substrate concentration, temperature and pH tolerance, operational stability, product inhibition, transgalactosylation performance and catalyst configuration. This process-specific interpretation provides the basis for the application-oriented comparisons presented in the subsequent sections.

Values are reproduced as reported in the original publications and were not interconverted or recalculated when the information required for reliable conversion was unavailable. Only kinetic parameters determined with lactose as the substrate are included. NR, not reported. Because assay temperature, pH, enzyme preparation, kinetic model and reaction matrix differed among studies, the values should not be interpreted as a strict cross-study ranking. Where matrix-specific *Kₘ* or *Vₘₐₓ* values were not reported in the original studies, they were designated as NR and were not inferred from buffer-derived measurements.

Table 3 demonstrates that lactose-specific kinetic properties vary markedly even among *β*-galactosidases belonging to the same GH family. Importantly, however, genuinely matrix-specific kinetic constants remain scarce. Most published *Kₘ* and *Vₘₐₓ* values have been determined using lactose in buffered model systems, whereas milk- and whey-based experiments have generally been used to quantify lactose conversion or GOS formation rather than to derive complete kinetic parameters. Recent work with BgaPw from *Paenibacillus wynnii* is particularly notable because lactose kinetics were evaluated both in Novo buffer and in a milk-based matrix, yielding *Kₘ* values of 0.34 ± 0.084 and 0.63 ± 0.045 mM, respectively, at 8 °C. In a separate milk-conversion experiment, BgaPw achieved essentially complete lactose hydrolysis after 72 h at 8 °C. Its transgalactosylation capacity was demonstrated separately using 400 g L^−1^ lactose in buffer, yielding approximately 144 g L^−1^ GOS after 24 h (36.0% yield) (Lutz-Wahl et al., 2024). Recombinant *S. thermophilus* LacZ was applied directly to whey permeate; at an initial lactose concentration of 205 g L^−1^, the maximum GOS yield reached approximately 50% of total sugar mass at ~90% lactose conversion at 50 °C (Geiger et al., 2016). In addition, recombinant *β*-galactosidase from Marinomonas sp. ef1 achieved lactose-hydrolysis yields of 82% in skimmed milk at 4 °C and 94% in cheese whey permeate at 50 °C (Bianchi et al., 2024). Consequently, unavailable matrix-specific *Kₘ* or *Vₘₐₓ* values were not estimated from buffer-derived measurements, and kinetic parameters should be interpreted together with matrix-specific conversion, operational stability, and process conditions rather than used as stand-alone indicators of industrial performance.

### 3.5 Physicochemical stability

#### 3.5.1 pH and temperature dependence

Physicochemical stability is a major determinant of *β*-galactosidase performance because catalytic activity and operational lifetime depend strongly on pH and temperature. However, optimum activity should not be interpreted as equivalent to process suitability: an enzyme may display a high instantaneous activity at its temperature optimum but lose activity rapidly during prolonged operation. Consequently, pH and temperature optima should be considered together with thermal half-life, matrix stability and the intended processing regime.

Recombinant bacterial *β*-galactosidases span a broad range of pH and temperature optima. For example, the LacLM *β*-galactosidase from *L. helveticus* DSM 20075 showed maximal lactose-hydrolysing activity at pH 6.5 and 60 °C, whereas its stability decreased markedly as the incubation temperature increased (Kittibunchakul et al., 2019). In contrast, the recently characterized BgaPw from *P. wynnii* exhibited a pH optimum of 7.0 and a temperature maximum of 40 °C but retained substantial activity during prolonged storage at low temperature, making it particularly relevant to refrigerated milk processing (Lutz-Wahl et al., 2024).

These examples illustrate that there is no universally optimal pH–temperature window for industrial *β*-galactosidases. Enzymes intended for refrigerated lactose hydrolysis require high activity and stability at low temperatures, whereas catalysts used for concentrated-lactose GOS synthesis may benefit from operation at higher temperatures because of increased lactose solubility and reduced microbial contamination. Therefore, the appropriate physicochemical profile depends on the intended application rather than on the highest reported activity optimum alone.

#### 3.5.2 Thermal and operational stability

Temperature optima alone provide limited information on industrial robustness. Operational stability is better assessed through residual activity, half-life and performance during prolonged reaction or storage under process-relevant conditions. This distinction is particularly important for dairy applications, where enzyme exposure may range from several hours during batch lactose conversion to extended reuse in immobilized or continuous systems.

The recombinant *β*-galactosidase from *L. helveticus* DSM 20075 illustrates the difference between catalytic optimum and thermal stability. Although maximal lactose-hydrolysing activity occurred at 60 °C, the reported activity half-life decreased from approximately 216 h at 30 °C to 5 h at 40 °C and 0.33 h at 50 °C; addition of 1 mM MgCl₂ substantially increased thermal stability, extending the half-life to 72 h at 40 °C and 3 h at 50 °C (Kittibunchakul et al., 2019).

Conversely, the cold-active BgaPw from *P. wynnii* showed half-lives of 77 d at 8 °C and 18 d at 30 °C, demonstrating that a relatively moderate temperature optimum can coexist with exceptional operational stability under refrigerated processing conditions (Lutz-Wahl et al., 2024). Matrix composition can also influence stability. Recombinant *S. thermophilus* LacZ was more stable in whey permeate than in phosphate buffer at 37 and 50 °C, indicating that process-matrix effects should be evaluated directly rather than inferred from buffer-based assays alone (Geiger et al., 2016).

Accordingly, application-oriented evaluation should prioritize operational half-life under the intended reaction conditions rather than temperature optimum alone. For refrigerated lactose hydrolysis, prolonged stability at 4–10 °C may be more valuable than maximal activity at 50–60 °C, whereas higher-temperature stability becomes more important for concentrated-lactose GOS synthesis and other processes operated at elevated temperatures.

### 3.6 Process factors affecting catalytic performance

#### 3.6.1 Product inhibition

Product inhibition can substantially affect lactose conversion, but its magnitude and mechanism are strongly enzyme-dependent. Galactose inhibits many microbial *β*-galactosidases through competition at or near the substrate-binding region, whereas glucose generally exerts a weaker effect; however, this behaviour is not universal. Consequently, product tolerance should be determined experimentally for individual enzymes rather than inferred from GH family or source organism alone.

Considerable variation has been reported among bacterial *β*-galactosidases. The LacLM *β*-galactosidase from *Lactobacillus sakei* Lb790 showed stronger inhibition by galactose than by glucose, with reported *Ki* values of 180 and 475 mM, respectively (Iqbal et al., 2011). In contrast, the recombinant BcGalB from *Bacillus coagulans* MA-13 showed measurable inhibition by both lactose-hydrolysis products, with glucose exerting a stronger effect than galactose under the tested conditions (Aulitto et al., 2021).

A particularly important recent exception is BgaPw from *P. wynnii*. Rather than being inhibited, the enzyme was activated by both galactose and glucose, with activity increases of up to 144% under the investigated conditions. The enzyme also achieved essentially complete lactose hydrolysis in milk at 8 °C, indicating that product tolerance can translate directly into favourable matrix-level performance (Lutz-Wahl et al., 2024).

These contrasting behaviours demonstrate that product inhibition should be treated as an enzyme-specific selection parameter. For high-conversion lactose hydrolysis, low sensitivity to accumulating galactose and glucose is particularly desirable because product concentrations increase progressively as the reaction approaches completion. For GOS synthesis, product effects should additionally be considered together with secondary hydrolysis of newly formed oligosaccharides.

#### 3.6.2 Effect of lactose concentration

Lactose concentration is a major process variable governing the partitioning between hydrolysis and transgalactosylation. At relatively low lactose concentrations, water is an efficient acceptor of the galactosyl–enzyme intermediate and hydrolysis predominates. Increasing lactose concentration increases the availability of carbohydrate acceptors and generally favours transgalactosylation, although the magnitude of this effect remains enzyme-specific.

This concentration dependence creates an important distinction between the two principal industrial applications considered in this review. Direct lactose hydrolysis in milk and liquid whey typically involves substantially lower lactose concentrations than GOS manufacture, whereas GOS synthesis generally relies on concentrated lactose feeds. A recent systematic review and meta-analysis of 104 studies published between 2018 and 2024 identified approximately 30–50% (w/v) lactose as the range most frequently associated with favourable GOS biosynthesis, together with reaction temperatures of approximately 40–60 °C under enzyme-specific pH conditions (Costa et al., 2025).

Recent bacterial examples support this process distinction. BgaPw from *P. wynnii* was evaluated for lactose hydrolysis directly in milk, whereas GOS synthesis was performed using 400 g/L lactose, yielding approximately 144 g/L GOS after 24 h, corresponding to a reported yield of 36% (Lutz-Wahl et al., 2024).

Similarly, recombinant *S. thermophilus β*-galactosidase converted whey permeate containing 50 g/L lactose predominantly toward hydrolysis, whereas increasing the initial lactose concentration to 205 g/L substantially increased GOS formation, reaching approximately 50% of total sugars at high lactose conversion (Geiger et al., 2016).

High substrate concentration therefore should not be interpreted simply as a means of increasing reaction rate. It changes reaction selectivity and introduces additional process constraints, including increased viscosity and mass-transfer limitations. Accordingly, lactose concentration should be selected according to the intended objective: relatively dilute dairy matrices for efficient lactose removal versus concentrated lactose streams for maximizing transgalactosylation and GOS production.

## 4 Recombinant production of bacterial *β*-galactosidases

### 4.1 Gene discovery and cloning strategies

Efficient recombinant production begins with the identification of *β*-galactosidase genes whose properties are compatible with the intended process, including lactose hydrolysis, transgalactosylation, temperature and pH tolerance, product inhibition and recombinant producibility. Genome sequencing, metagenomic screening and sequence-based mining have substantially expanded the diversity of bacterial *β*-galactosidases available for characterization. However, gene identification alone does not establish industrial suitability; candidate enzymes must subsequently be evaluated using lactose as the substrate and, where possible, under application-relevant matrix and process conditions. Following candidate selection, genes are cloned or synthesized for heterologous expression, with host-dependent optimization used where necessary to improve soluble or secreted production. Expression-vector architecture, promoter strength, signal peptide selection and gene dosage may substantially affect recombinant yield and therefore need to be optimized together with the host rather than considered independently (Movahedpour et al., 2022; Király et al., 2025). This strategy has been successfully applied to hydrolytically active *Bacillus licheniformis* T5 and *Bacillus paralicheniformis* T7, demonstrating the value of genomic resources for identifying promising industrial enzyme candidates (Mussakhmetov et al., 2024, 2026).

### 4.2 Recombinant expression systems

#### 4.2.1 Escherichia coli

*Escherichia coli* is the most widely used host for recombinant production of bacterial *β*-galactosidases because of its rapid growth, simple cultivation, well-established genetics and high recombinant protein yield. Numerous bacterial GH2 and GH42 *β*-galactosidases have been successfully expressed in *E. coli*, making it the predominant platform for laboratory-scale enzyme production, biochemical characterization and protein engineering (Li et al., 2025). Expression in *E. coli* has been successfully applied to numerous bacterial *β*-galactosidases, including enzymes from *L. helveticus*, *L. sakei, Lactobacillus plantarum, Lactobacillus fermentum* and *Bifidobacterium bifidum*, which have subsequently been characterized for lactose hydrolysis and GOS synthesis (Iqbal et al., 2010, 2011; Liu et al., 2011; Kittibunchakul et al., 2019; Füreder et al., 2020). These studies demonstrate that *E. coli* remains the preferred platform for laboratory-scale enzyme characterization owing to its high productivity and well-established molecular toolbox. Although *E. coli* enables efficient recombinant protein production, overexpression may result in inclusion body formation and reduced protein solubility. These limitations are commonly addressed through codon optimization, low-temperature induction and co-expression of molecular chaperones (Movahedpour et al., 2022; İncir and Kaplan, 2024). Recombinant *β*-galactosidases expressed in *E. coli* are most commonly produced using T7 promoter-based pET vectors, which provide strong inducible expression and high protein yields. Alternative systems, including pBAD vectors, enable tighter regulation of gene expression and are useful for proteins susceptible to inclusion body formation (Krefft et al., 2022; Zulkifly et al., 2023). However, because most recombinant *β*-galactosidases produced in *E. coli* are recovered intracellularly, cell disruption and clarification are required before downstream processing, which should be considered when translating laboratory expression systems to industrial enzyme manufacture.

#### 4.2.2 Komagataella phaffii (Pichia pastoris)

*Komagataella phaffii* is one of the most important eukaryotic expression hosts for industrial enzyme production because it combines high cell-density cultivation with efficient secretion of recombinant proteins. Secretory expression simplifies downstream purification, while proper protein folding and scalability make this yeast suitable for commercial enzyme manufacturing (Ford, 2026).

Importantly, *K. phaffii* has been experimentally validated for the production of recombinant bacterial *β*-galactosidases. The heterodimeric LacLM *β*-galactosidase from *Lactobacillus crispatus* B470 was functionally expressed and secreted in Pichia pastoris GS115. Two recombinant strains produced extracellular *β*-galactosidase activities of 24.5 and 31.0 U mL^−1^, respectively, and the secreted enzyme exhibited optimum activity at 50 °C and pH 5.5–6.5 (Nie et al., 2013). This study provides direct evidence that a bacterial LacLM-type *β*-galactosidase can be functionally assembled and secreted using a eukaryotic recombinant production system. The industrial potential of *K. phaffii* has been demonstrated by the successful production of recombinant bovine and camel chymosin for cheese manufacture. Recombinant camel chymosin exhibited high milk-clotting activity, low proteolytic activity and excellent performance in dairy applications, highlighting the suitability of this expression system for large-scale production of food enzymes (Akishev et al., 2021, 2022, 2023).

Recombinant proteins in *K. phaffii* are most frequently expressed using pPICZ-derived vectors under the control of the AOX1 promoter, enabling high-level methanol-inducible expression together with efficient extracellular secretion (Ford, 2026).

Compared with bacterial hosts, *K. phaffii* offers the additional advantage of efficient extracellular secretion, which substantially simplifies downstream purification. Consequently, this system is particularly attractive for scalable production of food enzymes because extracellular secretion can simplify downstream recovery; however, regulatory suitability remains dependent on the specific production strain, genetic construct, manufacturing process, and intended use.

#### 4.2.3 Bacillus subtilis

*Bacillus subtilis* is an attractive production host for food-enzyme biotechnology owing to its capacity for extracellular protein secretion, which can simplify downstream recovery and reduce processing requirements. Its GRAS status further supports applications involving food enzymes. Nevertheless, endogenous extracellular proteases may decrease recombinant protein stability, and therefore protease-deficient strains together with optimized fermentation strategies are commonly employed to improve recombinant protein yield (Pozo et al., 2026).

Direct experimental evidence is available for recombinant bacterial *β*-galactosidase production in this host. The BMG *β*-galactosidase from *Bacillus megaterium* YZ08 was secretorily expressed in *B. subtilis* WB800 using the pMA5 expression vector and WapA signal peptide. The recombinant enzyme showed unusual tolerance to glucose and galactose and produced 278 g L^−1^ GOS from 600 g L^−1^ lactose within 12 h, demonstrating the feasibility of coupling secretory recombinant production in *B. subtilis* with high-solids GOS synthesis (Zhao et al., 2018).

#### 4.2.4 Lactococcus lactis

*Lactococcus lactis* is a food-grade expression host widely used in dairy biotechnology. Its absence of endotoxins and compatibility with food production make it particularly attractive for enzymes intended for direct food applications, although recombinant protein yields are generally lower than those achieved in *E. coli* (Frelet-Barrand, 2022; Liu et al., 2022). Because *L. lactis* is naturally associated with dairy fermentations, recombinant enzymes produced in this host are particularly suitable for applications involving direct food processing. Furthermore, tightly regulated expression systems, such as the NICE system, enable controlled production of recombinant proteins while minimizing metabolic burden on the host cell (Frelet-Barrand, 2022).

Recombinant *β*-galactosidase production has also been demonstrated directly in *L. lactis* (Yin et al., 2018). A *β*-galactosidase gene from *Lactobacillus delbrueckii* subsp. bulgaricus was expressed in *L. lactis* MG1363 using the pMG36e-based system, and incorporation of the Usp45 secretion signal enabled extracellular enzyme production. The recombinant strain exhibited measurable *β*-galactosidase activity in both cellular and extracellular fractions, demonstrating the feasibility of *L. lactis* as a host for production and secretion of a heterologous bacterial *β*-galactosidase (Zhang et al., 2012).

#### 4.2.5 Expression hosts and vectors

Additional hosts, including *Lactiplantibacillus plantarum*, *Corynebacterium glutamicum* and filamentous fungi, have also been explored for recombinant *β*-galactosidase production. Although less commonly used, these systems may offer advantages such as improved secretion, enhanced protein folding or suitability for specific industrial processes (Merkel et al., 2022; Ding and Ye, 2023; Shi et al., 2024).

The efficiency of recombinant *β*-galactosidase production depends not only on the choice of the expression host but also on the design of the expression vector. Expression vectors regulate gene transcription, translation and protein production and typically contain a promoter, ribosome-binding site, multiple cloning site, selectable marker and transcription terminator. Affinity tags, particularly His₆-tags, are frequently incorporated to facilitate downstream purification by immobilized metal affinity chromatography (Sajid et al., 2024).

Different expression hosts require dedicated vector systems. In *E. coli*, the T7 promoter-based pET vectors remain the most widely used owing to their high expression efficiency, whereas pBAD vectors provide tightly regulated arabinose-inducible expression. For *Komagataella phaffii*, recombinant proteins are commonly produced using pPICZ vectors under the control of the AOX1 promoter, enabling high-level secretory expression. In *B. subtilis*, pHT vectors are frequently employed to combine extracellular secretion with GRAS status, while the NICE expression system is the standard platform for recombinant protein production in *L. lactis*. Alternative hosts, including Lactiplantibacillus plantarum and *C. glutamicum*, use host-specific expression systems that can be optimized for food-compatible production, secretion or enhanced soluble protein recovery (Krefft et al., 2022; Ding and Ye, 2023; Zulkifly et al., 2023).

Table 4 summarizes the principal recombinant expression platforms together with their corresponding vector systems, highlighting differences in protein yield, secretion capability, regulatory status and industrial applicability. Whereas *E. coli* remains the preferred host for laboratory-scale enzyme production because of its high expression level and extensive molecular toolbox, secretory hosts such as *K. phaffii*, *B. subtilis* and *L. lactis* provide important advantages for industrial manufacturing by simplifying downstream processing and improving food compatibility.

**Table 4:** Comparison of recombinant expression systems experimentally used for bacterial *β*-galactosidase production.

Host	Representative expression system	Representative *β*-galactosidase	Expression mode	Main advantages	Major limitations	Experimental/process relevance	References
*Escherichia coli*	T7/pET-based recombinant expression; GroEL/GroES co-expression where applicable	LacLM from *L. helveticus* DSM 20075; *β*-gal I/II from *B. breve* DSM 20213	Mainly intracellular	High recombinant production; chaperone-assisted expression applicable	Inclusion bodies; cell disruption and clarification required	~26 kU L^−1^ activity reported for *L. helveticus* LacLM; purified recombinant enzymes used for biochemical characterization and GOS studies	Arreola et al. (2014), Kittibunchakul et al. (2019)
*Komagataella phaffii* (*Pichia pastoris*)	*P. pastoris* GS115 expression system	LacLM from *L. crispatus* B470	Extracellular	Secretion facilitates extracellular enzyme recovery	Longer cultivation; secretion must be optimized for individual enzymes	Up to 31.0 U mL^−1^ extracellular *β*-galactosidase activity in culture supernatant	Nie et al. (2013)
*Bacillus subtilis*	Secretory recombinant expression system	BMG from *B. megaterium* YZ08	Extracellular	Efficient secretory production; avoids intracellular enzyme recovery	Extracellular proteolysis; secretion optimization required	278 g L^−1^ GOS produced from 600 g L^−1^ lactose within 12 h; lactose completely consumed	Zhao et al. (2018)
*Lactococcus lactis* MG1363	pMG36e-usp-lacZ with Usp45 signal peptide	*β*-galactosidase from *L. delbrueckii* subsp. *bulgaricus*	Cellular + secreted fraction	Food-associated Gram-positive host; secretion demonstrated	Lower recombinant production than major industrial secretion hosts	*β*-Galactosidase activity 0.225 U mL^−1^; secretion rate 10%; enzyme detected in both supernatant and cell fractions	Zhang et al. (2012)
*Lactiplantibacillus plantarum* WCFS1	Plasmid-based recombinant expression	LacZ from *S. thermophilus* DSM 20259	Intracellular	Food-grade host; direct relevance to dairy bioprocessing	Cell disruption required for intracellular enzyme	~11,000 U L^−1^ culture; ~170 mg recombinant enzyme; crude enzyme applied to whey permeate; ~50% GOS of total sugars from 205 g L^−1^ lactose at 50 °C and 90% lactose conversion	Geiger et al. (2016)

Table 4 compares the principal recombinant hosts used for bacterial *β*-galactosidase production together with representative expression systems, secretion modes, major advantages and limitations, and experimentally demonstrated process relevance. The comparison demonstrates that no universal expression platform exists and that host selection should be guided by the intended application, recombinant protein characteristics and downstream processing requirements.

*Escherichia coli* remains the predominant host for laboratory-scale production owing to its rapid growth, well-established molecular toolbox and capacity for high-level recombinant protein expression. Nevertheless, intracellular production may result in inclusion body formation and requires cell disruption and clarification during downstream processing. In contrast, secretory production in *K. phaffii* and *B. subtilis* can facilitate recovery of recombinant *β*-galactosidases from the culture supernatant and thereby simplify downstream processing.

*Lactococcus lactis* represents an attractive alternative for dairy biotechnology because it is a food-associated Gram-positive host and has been successfully used for heterologous bacterial *β*-galactosidase production and secretion. Tightly regulated systems such as NICE are available for recombinant protein production in *L. lactis*, although individual *β*-galactosidase studies have also employed other expression systems. Lactiplantibacillus plantarum has likewise been used for recombinant *β*-galactosidase production and subsequent evaluation in dairy-related applications.

Overall, Table 4 highlights that the choice of recombinant production platform represents a compromise among expression level, soluble protein recovery, secretion efficiency, downstream-processing requirements and regulatory suitability. Consequently, expression-host selection should be considered together with the intended catalyst format and industrial application rather than being based solely on recombinant protein yield.

### 4.3 Regulatory considerations for recombinant food-enzyme production

Regulatory suitability of recombinant *β*-galactosidase production depends not only on the expression host but also on the specific production strain, introduced genetic material, selectable markers, manufacturing process, final enzyme preparation and intended conditions of use. Consequently, designations such as «GRAS», «food-grade» or «QPS» should not be interpreted as automatic authorization of every recombinant enzyme produced using a particular microorganism.

In the European Union, food enzymes are regulated under Regulation (EC) No 1332/2008 and are subject to safety assessment by the European Food Safety Authority (EFSA). The assessment considers the source and identity of the enzyme, characteristics of the production microorganism, genetic modifications where applicable, manufacturing process, enzyme preparation, intended uses, dietary exposure, toxicological information and potential allergenicity. For enzymes produced using genetically modified microorganisms, particular attention is given to characterization of the production strain and genetic modification and, where applicable, demonstration of the absence of viable production organisms and recombinant DNA in the final enzyme preparation. Authorization additionally requires a demonstrated technological need and that the proposed use does not mislead consumers (U.S. Food and Drug Administration, 2010; Lambré et al., 2021).

In the United States, enzyme preparations used in food may be regulated as food additives or may be considered Generally Recognized as Safe (GRAS) under their intended conditions of use. Evaluation of microbial enzyme preparations considers the identity and characteristics of the enzyme and production organism, genetic modifications, manufacturing process, composition and purity of the enzyme preparation, intended technical effect and conditions of use. Manufacturing is expected to follow current good manufacturing practice, while materials used during fermentation and downstream processing should be suitable for food production. Thus, GRAS status is specific to the substance and its intended conditions of use and should not be inferred solely from the taxonomic identity of the production host U.S. Food and Drug Administration (2010).

International specifications provide an additional reference framework for food-enzyme quality and safety. The FAO/WHO Joint Expert Committee on Food Additives (JECFA) specifications for enzyme preparations and the Food Chemicals Codex establish criteria relevant to identity, purity and microbiological quality and are frequently considered in the safety evaluation and commercial manufacture of food-enzyme preparations (FAO/WHO JECFA, 2006; U.S. Food and Drug Administration, 2010). Although regulatory pathways differ among jurisdictions, they converge on several common requirements: characterization of the production organism and genetic construct, controlled manufacturing under appropriate quality systems, assessment of potential contaminants and toxicological hazards, defined enzyme identity and activity, and demonstration of safety under the intended conditions of use FAO/WHO JECFA (2006), Lambré et al. (2021), U.S. Food and Drug Administration (2010).

These regulatory considerations have direct implications for recombinant-production strategy. For industrial *β*-galactosidases, host and vector selection should minimize unnecessary antimicrobial-resistance markers and facilitate control or removal of production organisms, recombinant DNA and process-derived impurities where required (Lambré et al., 2021). Secretory hosts may offer an additional manufacturing advantage by reducing cell-disruption requirements, although secretion itself does not establish regulatory acceptability. Regulatory compatibility should therefore be considered together with recombinant yield, secretion efficiency, downstream-processing requirements and intended food application from the earliest stages of process development.

### 4.4 Factors governing soluble expression

For intracellular recombinant production, high soluble expression is important for maximizing recovery of catalytically active *β*-galactosidase because protein aggregation into inclusion bodies substantially reduces enzyme recovery and increases downstream processing costs. Protein solubility is influenced by multiple factors, including codon usage, promoter strength, induction temperature, inducer concentration, expression rate, host strain and cultivation conditions (Movahedpour et al., 2022).

Among these parameters, induction temperature is one of the most influential. Lower induction temperatures, typically 16-25 °C in *E. coli,* reduce the rate of protein synthesis, allowing more efficient folding and decreasing inclusion body formation. Similarly, reduced IPTG concentrations and controlled promoter induction frequently improve the proportion of soluble recombinant enzyme. Additional strategies, including codon optimization, co-expression of molecular chaperones (GroEL/GroES and DnaK/DnaJ systems) and fermentation optimization, further enhance soluble protein yield and catalytic activity (İncir and Kaplan, 2024; Zhou et al., 2024; Király et al., 2025). For example, co-expression of GroEL/GroES was required to improve recombinant production of *β*-gal I and *β*-gal II from *B. breve* DSM 20213 (Arreola et al., 2014).

### 4.5 Purification strategies

For laboratory biochemical characterization, recombinant *β*-galactosidases are frequently produced with N- or C-terminal His-tags and purified by immobilized metal affinity chromatography (IMAC), with additional chromatographic steps used where higher purity is required (Arreola et al., 2014; Kittibunchakul et al., 2019). These approaches are particularly useful when highly purified enzyme is required for kinetic, structural or mechanistic characterization. However, affinity-based purification and multiple chromatographic polishing steps may increase downstream complexity and cost and therefore should not be regarded as the default strategy for large-scale food-enzyme manufacture.

At industrial scale, downstream processing should minimize the number and cost of unit operations while maintaining enzyme recovery, activity and food compatibility. For intracellular *β*-galactosidases, a scalable workflow may comprise biomass recovery by centrifugation or filtration, cell disruption, clarification, selective precipitation or fractionation, followed by ultrafiltration/diafiltration (UF/DF) for enzyme concentration, buffer exchange and removal of low-molecular-weight process components. For secreted enzymes, cell disruption can be avoided, allowing clarified fermentation supernatant to proceed directly to concentration and formulation. Membrane-based processing is particularly attractive because UF/DF can concentrate and desalt enzyme preparations without requiring affinity ligands. The applicability of this approach to *β*-galactosidase processing has been demonstrated experimentally: ultrafiltration of a crude enzyme extract using a 50 kDa polyethersulfone membrane resulted in up to 7.1-fold volume concentration, while diafiltration enabled salt removal after precipitation and ion-exchange chromatography with enzyme recovery above 93.4% (Lemes et al., 2023). Although this study employed *β*-galactosidase from *Kluyveromyces marxianus*, it demonstrates the practical applicability of membrane-based concentration and desalting as downstream unit operations beyond affinity chromatography.

The required degree of purification should ultimately be determined by the intended catalyst format and food application rather than by analytical purity alone. Crude or partially purified enzyme preparations may be appropriate when process-derived impurities and residues of the production organism are adequately controlled and the preparation complies with applicable food-safety requirements. This principle is illustrated by recombinant *S. thermophilus β*-galactosidase, for which a crude cell-free preparation was applied directly to whey permeate for lactose conversion and GOS production (Geiger et al., 2016). Where higher purity is required, conventional fractionation or chromatographic polishing, including ion-exchange chromatography, can be incorporated after initial recovery and concentration (Lemes et al., 2023). Thus, precipitation, membrane concentration/diafiltration and, where required, conventional chromatographic fractionation provide scalable alternatives or complements to His-tag/IMAC purification. Selection of the downstream sequence should therefore balance enzyme recovery, throughput, consumable requirements, number of unit operations, food compatibility, regulatory requirements and overall process economics.

The availability of efficient recombinant production and purification strategies has considerably expanded the industrial application of carbohydrate-active enzymes beyond *β*-galactosidases. Similar recombinant expression platforms have recently been employed for the production of xylanases, amylases and other glycoside hydrolases. For example, recombinant xylanase from *Bacillus sonorensis* T6 was successfully applied to the hydrolysis of cereal straw for xylooligosaccharide production, demonstrating the versatility of recombinant enzyme technologies in sustainable biorefinery applications (Shamsiyeva et al., 2026). Figure 3 presents the integrated workflow for the development of recombinant bacterial *β*-galactosidases, encompassing gene discovery, cloning, heterologous expression, downstream processing, biochemical characterization, industrial validation and final biotechnological applications.

**Figure 3:**
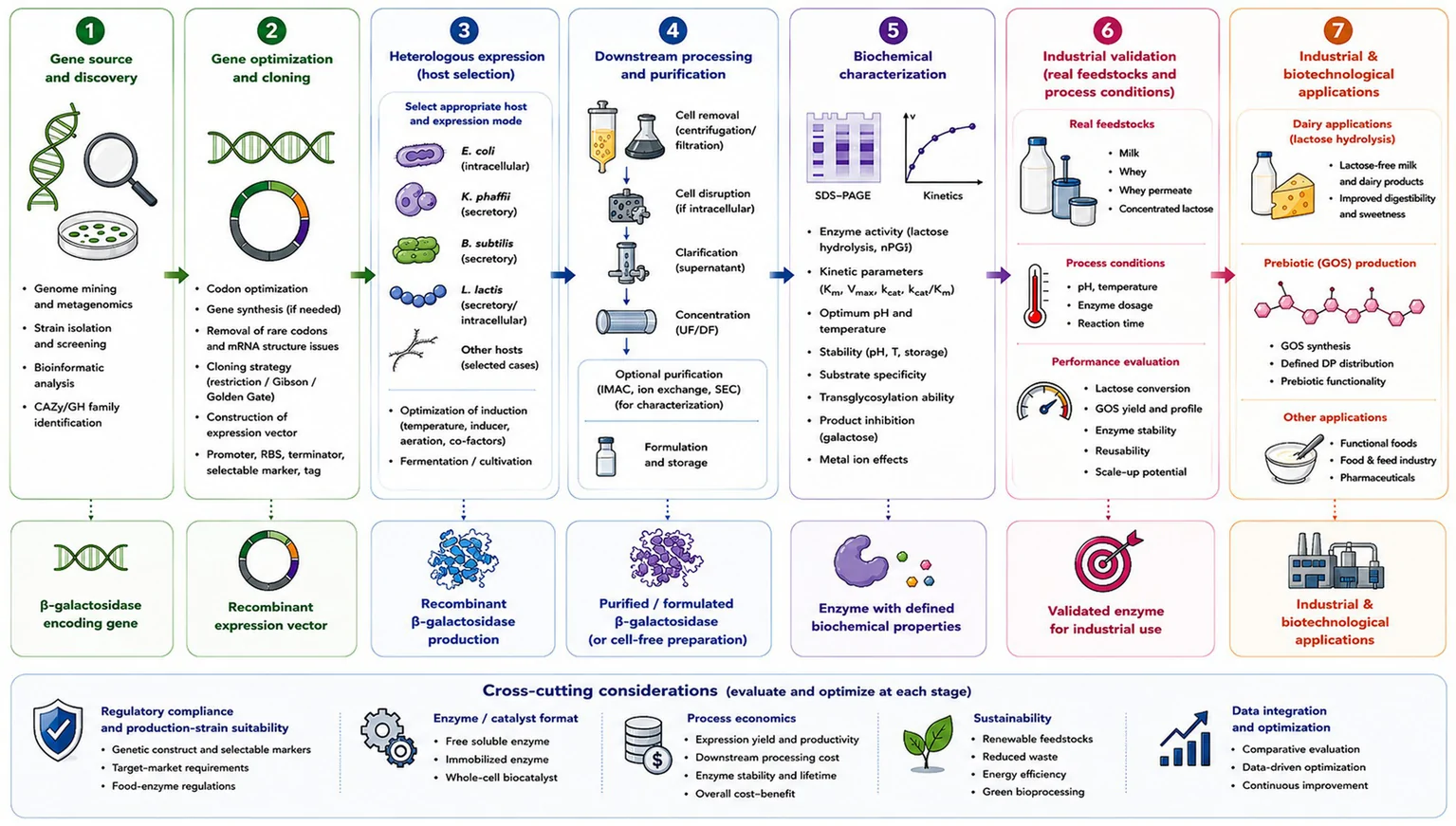
Integrated workflow for the discovery, recombinant production, characterization and industrial validation of bacterial *β*-galactosidases.

The workflow integrates gene discovery and construct design with expression-host selection, downstream processing, biochemical characterization and validation under application-relevant conditions. Industrial validation includes testing in real dairy matrices (milk, whey and whey permeate) and concentrated lactose feeds, depending on whether the target application is lactose hydrolysis or GOS production. Catalyst format, operational stability, reusability, regulatory compatibility and process economics are considered as cross-cutting criteria for industrial implementation.

Figure 3 summarizes an application-oriented workflow for translating newly identified bacterial *β*-galactosidases from gene discovery to industrial implementation. Unlike laboratory workflows focused primarily on recombinant expression and analytical purification, industrial development additionally requires scalable downstream processing, appropriate catalyst formulation and validation under process-relevant conditions. Importantly, the validation stage should distinguish between low- to moderate-lactose dairy matrices used for lactose hydrolysis and concentrated lactose feeds used for GOS production.

### 4.6 Current bottlenecks

Despite substantial advances in recombinant expression technology, efficient production of bacterial *β*-galactosidases remains challenging. Major bottlenecks include poor protein solubility, inclusion body formation, low recombinant protein yield, limited enzyme stability and high purification costs (Liu M. et al., 2025). For industrial food-enzyme manufacture, these bottlenecks additionally include downstream recovery costs, dependence on affinity purification, limited secretion efficiency and the need to demonstrate performance in real process matrices.

Current optimization strategies include codon optimization, promoter engineering, host strain improvement, fermentation optimization, protein engineering and artificial intelligence-assisted enzyme design. Together, these approaches have significantly improved soluble protein production, enzyme stability and overall recombinant expression efficiency and are expected to accelerate the development of next-generation *β*-galactosidases with enhanced catalytic and industrial performance (Király et al., 2025).

Collectively, these optimization strategies determine not only recombinant protein yield but also enzyme quality, process scalability and industrial feasibility. They therefore provide the technological foundation for the rational comparative evaluation and selection of bacterial *β*-galactosidases for industrial applications, as discussed in the following chapter.

## 5 Comparative assessment of recombinant bacterial *β*-galactosidases

### 5.1 Comparative catalytic and process-relevant characteristics

Meaningful comparison of recombinant bacterial *β*-galactosidases requires a clear distinction between lactose-specific catalytic kinetics and complementary biochemical or process-related properties. Lactose-derived kinetic parameters, including *K_m_, V_max_, k_cat_,* and *k_cat_/K_m_*, are therefore evaluated separately in Table 3. Even when lactose is used as the substrate, direct comparison of these parameters requires caution because reported values depend on enzyme preparation, assay matrix, substrate concentration range, temperature, pH, kinetic model, and other experimental conditions (Singh Purewal and Kaur, 2024; Akishev et al., 2025).

Table 5 consequently focuses on experimentally reported properties that influence enzyme performance under processing conditions rather than on heterogeneous specific-activity values. These properties include optimum pH and temperature, operational stability, product inhibition or tolerance, effects of ions and additives, recombinant production format, and, where available, performance in lactose-containing matrices. Separating these characteristics from lactose-specific kinetic parameters avoids constructing an artificial ranking from activity values obtained using different substrates, units, enzyme preparations, and assay protocols.

**Table 5:** Experimentally reported process-relevant properties and application evidence for selected bacterial *β*-galactosidases.

Source/enzyme	Recombinant host/enzyme format	Assay substrate or matrix	Optimal temperature (°C)	Optimal pH	Operational stability	Product inhibition/ tolerance	Effects of ions/additives	Application-relevant evidence	References
*Lactobacillus plantarum* WCFS1	Recombinant enzyme; heterologous expression in *Escherichia coli*	oNPG; lactose; concentrated lactose solution	55 (oNPG), 60 (lactose)	7.5 (oNPG); 7.0 (lactose)	Stable over approximately pH 6.5–8.0; reported stability at moderate processing temperatures	Galactose inhibits activity	Mn^2+^, Mg^2+^, K^+^ and Na^+^ stimulate activity; Mg^2+^ contributes to stability	GOS synthesis demonstrated from 600 mM lactose; maximum GOS yield ≈41% (w/w of total sugars) at ≈85% lactose conversion	Iqbal et al. (2010)
*Lactobacillus sakei* Lb790	Recombinant heterodimeric GH2 enzyme expressed in *E. coli*	oNPG; lactose; concentrated lactose solution	55	6.0–7.5; substrate-dependent optimum reported	Mg^2+^ supports enzyme stability/activity	Competitive inhibition by galactose, K_i_ = 180 mM, and glucose, K_i_ = 475 mM; comparatively moderate product inhibition	Activated/stabilized by Mg^2+^	GOS synthesis demonstrated at 215 g/L lactose; maximum GOS yield 41% (w/w) at 77% lactose conversion; products mainly DP2–DP4, predominantly *β*-(1 → 6)-linked	Iqbal et al. (2011)
*Lactobacillus delbrueckii* subsp. *bulgaricus* DSM 20081 *β*-galactosidase	Recombinant homodimeric GH2 enzyme expressed in food-grade *L. plantarum*	oNPG; lactose; concentrated lactose solution	55–60	broad activity range, approximately pH 6.0–9.0	Reported as relatively stable under near-neutral processing conditions	Galactose inhibition reported; enzyme retains substantial transgalactosylation capacity during lactose conversion	Na^+^, K^+^ and Mg^2+^ enhance activity; Ca^2+^ and Zn^2+^ can inhibit	GOS production demonstrated from 205 g/L lactose; approximately 50% maximum GOS yield with high lactose conversion under the reported reaction conditions	Nguyen et al. (2012)
*Lactobacillus helveticus* DSM 20075 LacLM	Recombinant enzyme expressed in *E. coli*; additional recombinant expression systems investigated	oNPG; lactose	55 (oNPG); approximately 60 (lactose)	approximately 6.5; activity retained across a broader near-neutral range	Addition of 1 mM MgCl₂ markedly enhanced thermal stability; reported activity half-life increased from 5 to 72 h at 40 °C and from 0.33 to 3 h at 50 °C	Product response is substrate- and assay-dependent; direct industrial-matrix product-inhibition data remain limited	Na^+^ and K^+^ can enhance activity; Mg^2+^ strongly affects stability; Mn^2+^, Ca^2+^, Zn^2+^ and Cu^2+^ can reduce activity depending on conditions	Characterized specifically for potential dairy application, but much of the biochemical evidence was obtained with purified enzyme in model systems rather than directly in milk or whey	Kittibunchakul et al. (2019)
*Limosilactobacillus fermentum* K4 LacLM	Recombinant heterodimeric enzyme expressed in *E. coli* Origami B (DE3)	oNPG; lactose; concentrated lactose solution	40 (oNPG); 45–50 (lactose)	7.0 (oNPG); 6.5 (lactose)	Broad pH optimum and stability around neutral pH	Product inhibition was not the principal focus; performance demonstrated at high lactose concentration	Activity stimulated by Na^+^, K^+^ and Mg^2+^; 15% ethanol also increased activity	GOS production demonstrated; maximum GOS accounted for approximately 37% of total sugars. Reported process conditions include high-solids lactose systems	Benavente et al. (2015)
*Pantoea anthophila β*-galactosidase B-gal42	Recombinant *β*-galactosidase; heterologous expression system	Lactose and whey; concentrated feed systems	60	7.0	Function retained under conditions used for concentrated lactose/whey conversion	Specific glucose/galactose inhibition constants not reported in the cited application study	Mg^2+^, Mn^2+^, Na^+^ and K^+^ reported to influence activity; ion effects depend strongly on concentration	Direct GOS synthesis demonstrated using both lactose and whey. At 300 g/L substrate, approximately 38% GOS yield was reported at ≈60% lactose conversion, with comparable product profiles for lactose and whey	Yañez-Ñeco et al. (2021)
*Lactobacillus plantarum β*-galactosidase	Recombinant enzyme expressed in *E. coli*; immobilization subsequently investigated	oNPG; concentrated lactose solution	40	9.0	Immobilization was investigated as a strategy to improve enzyme robustness and reuse	Glucose, galactose and high lactose concentrations can reduce activity	Ca^2+^ and Mg^2+^ reported to increase activity; Mn^2+^ inhibitory under the tested conditions	GOS synthesis reported from concentrated lactose; approximately 34% GOS yield has been reported at 400 g/L initial lactose in the associated process studies	Benavente et al. (2015)
*Lactobacillus bulgaricus* L3 *β*-galactosidase	Recombinant CBD-fusion enzyme expressed in *E. coli* and immobilized on cellulose	Concentrated lactose solution	45	near-neutral conditions	Immobilization enabled catalyst recovery/reuse and represents a process-relevant format beyond soluble purified enzyme	NR	Immobilization rather than soluble-enzyme additives was the principal process modification	Immobilized catalyst produced approximately 49% GOS from 400 g/L lactose within about 75 min under the reported conditions	Lu et al. (2012)
*Sulfolobus solfataricus β*-galactosidase expressed in *Lactococcus lactis* LM0230	Recombinant enzyme deployed in permeabilized whole cells	Concentrated lactose solution	85	thermophilic enzyme conditions	Whole-cell format provides catalyst containment and avoids complete purification	NR	Whole-cell permeability and thermal operating conditions are more relevant than metal-ion supplementation	Permeabilized recombinant whole cells generated approximately 197 g/L GOS from 400 g/L lactose after prolonged high-temperature conversion, illustrating an alternative catalyst format to purified soluble enzyme	Yu and O’Sullivan (2014)

Values are reported under the experimental conditions described in the cited studies and should not be interpreted as directly comparable performance rankings. Lactose-specific kinetic parameters (*K_m_*, *V_max_*, *k_cat_* and *k_cat_/K_m_*) are summarized separately in Table 3. Optimum temperature and pH values may depend on the assay substrate and reaction conditions. «Application-relevant evidence» distinguishes characterization in model buffer systems from experiments conducted with lactose-containing dairy matrices or concentrated lactose feeds. NR, not reported.

Published studies demonstrate substantial enzyme-to-enzyme variation, including among *β*-galactosidases assigned to the same glycoside hydrolase family. Taxonomic origin and GH-family membership can provide useful structural and mechanistic context, but neither is sufficient to predict process performance. Industrial suitability instead depends on the interaction between lactose-specific catalytic behaviour and operational characteristics under application-relevant conditions, including temperature and pH stability, susceptibility to glucose or galactose inhibition, compatibility with the processing matrix, and, for GOS manufacture, transgalactosylation performance at high lactose concentrations.

Many *β*-galactosidases from lactic acid bacteria exhibit maximum activity at approximately 40-60 °C and near-neutral pH, although substantial enzyme-specific variation has been reported. For example, some *Lactobacillus*-derived enzymes show optimal activity under neutral conditions, whereas the *β*-galactosidase characterized by Benavente et al. (2015) exhibited an alkaline optimum around pH 9.0. Such optimum values should not, however, be interpreted as direct indicators of industrial suitability because they are commonly determined in short-duration assays using chromogenic substrates rather than during prolonged lactose conversion in dairy matrices.

Product inhibition represents another important discriminator among candidate enzymes, particularly as glucose and galactose accumulate during lactose hydrolysis. The LacLM *β*-galactosidase from *L. delbrueckii* subsp. *bulgaricus* showed a reported galactose inhibition constant (K_i_) of 70.7 mM, whereas the enzyme from *L. sakei* Lb790 exhibited Ki values of 180 mM for galactose and 475 mM for glucose (Iqbal et al., 2011; Nguyen et al., 2012). Other *β*-galactosidases display markedly different responses to reaction products, demonstrating that product tolerance is an enzyme-specific property that should be determined under conditions representative of the intended application rather than inferred from taxonomic origin or GH-family assignment.

Metal ions and other medium components may likewise affect activity and operational stability. For recombinant *β*-galactosidase from *L. helveticus* DSM 20075, supplementation with 1 mM MgCl₂ substantially increased thermal stability, extending the reported activity half-life from 5 to 72 h at 40 °C and from 0.33 to 3 h at 50 °C (Kittibunchakul et al., 2019). However, responses to mono- and divalent ions vary considerably among bacterial *β*-galactosidases and may also depend on buffer composition and ion concentration. Metal-ion effects should therefore be treated as experimentally specific properties rather than generalized characteristics of a genus or GH family.

A further limitation of the available literature is that many recombinant bacterial *β*-galactosidases have been characterized predominantly using purified enzymes, synthetic substrates, and buffered reaction systems. Such experiments are essential for biochemical characterization but provide incomplete information about performance in industrial matrices, where lactose concentration, ionic composition, proteins, minerals, viscosity, water activity, and accumulating reaction products may alter apparent enzyme behaviour. Evidence obtained directly in milk, whey, whey permeate, or concentrated lactose feeds should therefore be given greater weight when evaluating industrial applicability.

Importantly, the relevant evaluation criteria differ between lactose hydrolysis and GOS manufacture. Hydrolysis of lactose in milk or whey generally requires effective catalytic performance under the temperature, pH, and compositional constraints of the dairy matrix, with product tolerance and operational stability becoming particularly important as conversion proceeds. In contrast, GOS synthesis is typically performed using substantially more concentrated lactose feeds, for which high transgalactosylation efficiency, tolerance to elevated substrate and product concentrations, control of secondary GOS hydrolysis, and the resulting oligosaccharide profile become major evaluation criteria. Consequently, these two applications should not be evaluated using a single activity-based ranking.

Overall, the available evidence indicates that no single bacterial taxonomic group or GH family consistently combines favourable lactose kinetics, operational stability, product tolerance, matrix compatibility and transgalactosylation performance. Comparative evaluation should therefore consider lactose-specific kinetic data together with experimentally demonstrated process-relevant properties and, whenever available, performance in the intended reaction matrix. Importantly, the limited availability of directly comparable data from milk, whey and other industrially relevant matrices currently restricts quantitative comparison of many recombinant *β*-galactosidases. These limitations should be considered when interpreting the multidimensional comparisons presented in the following sections.

### 5.2 Comparative recombinant production characteristics

Successful industrial application depends not only on catalytic performance but also on the feasibility of recombinant enzyme production at scale. Recombinant bacterial *β*-galactosidases differ considerably in expression host, intracellular or secreted production, soluble protein recovery, volumetric productivity, downstream-processing requirements, and compatibility of the production organism with food-enzyme manufacture. These factors can substantially influence production cost and process scalability independently of the intrinsic catalytic properties of the enzyme (Movahedpour et al., 2022; Király et al., 2025).

*Escherichia coli* remains the most frequently used heterologous host for the characterization of bacterial *β*-galactosidases because of its rapid growth, established genetic tools, and capacity for high-level recombinant expression (Movahedpour et al., 2022; Liu M. et al., 2025). However, expression yield in *E. coli* should not be equated directly with industrial production suitability. Recombinant proteins may accumulate intracellularly or form insoluble aggregates, requiring cell disruption and additional recovery steps, while laboratory-scale affinity purification using His-tags and immobilized-metal affinity chromatography (IMAC) is primarily appropriate for enzyme characterization rather than routine large-scale food-enzyme manufacture (Movahedpour et al., 2022; Liu M. et al., 2025).

Alternative expression platforms can address some of these limitations. *Bacillus subtilis* and related *Bacillus* hosts offer the possibility of extracellular protein secretion, potentially simplifying enzyme recovery, whereas *L. lactis* provides a food-associated bacterial production platform (Zhang et al., 2012; Zhao et al., 2018). The yeast *Komagataella phaffii* is also widely used for recombinant protein production because high-cell-density cultivation and secretory expression can support high volumetric productivity (Liu M. et al., 2025). Nevertheless, the suitability of each host depends on the individual *β*-galactosidase, secretion efficiency, protein folding, proteolytic stability, cultivation requirements, regulatory status of the production strain, and the intended downstream process. Therefore, expression-host selection should be evaluated using experimentally demonstrated enzyme production rather than inferred solely from the general characteristics of the host.

For industrial production, downstream processing must likewise be distinguished from purification strategies used for biochemical characterization. His-tag purification by IMAC, followed by techniques such as size-exclusion chromatography, is valuable for obtaining highly purified protein for kinetic and structural studies but is generally difficult to justify for bulk food-enzyme production because of resin cost, processing complexity, and scale. Industrially relevant strategies may instead involve extracellular secretion followed by biomass removal and concentration, membrane-based recovery, precipitation or other scalable fractionation procedures, depending on the required purity and intended application. Immobilized enzymes and whole-cell catalysts can further reduce purification requirements by allowing catalyst retention and reuse (Duan et al., 2023).

To examine how production characteristics interact with catalytic and process-related properties, representative bacterial *β*-galactosidases were compared across structural, catalytic, recombinant-production, and application-related variables (Figure 4). The heatmap is intended as an integrative visualization rather than as an absolute ranking of enzyme performance because the underlying literature data were generated under heterogeneous experimental conditions. Quantitative variables were standardized before visualization, whereas categorical variables were encoded separately. Missing values were retained as unavailable information rather than interpreted as zero activity or poor performance. Consequently, clustering should be interpreted as exploratory identification of enzymes with similar reported property profiles rather than as evidence of phylogenetic or mechanistic relationships.

**Figure 4:**
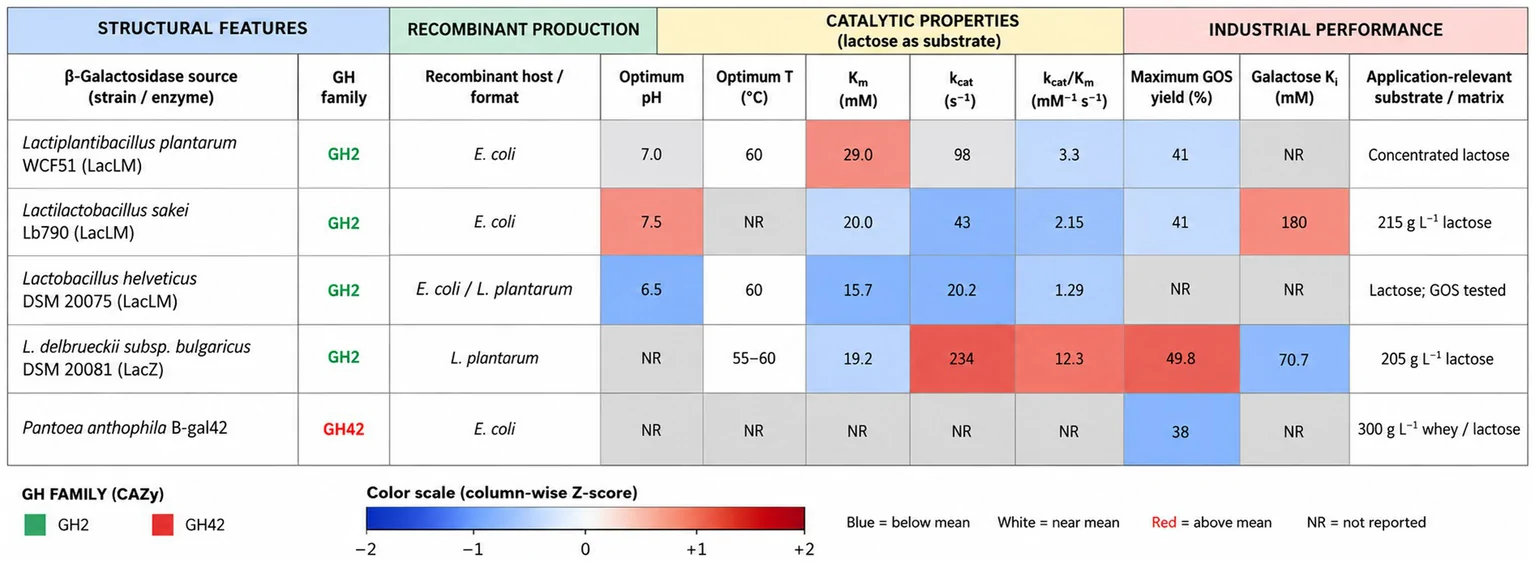
Integrated comparison of structural, catalytic, recombinant-production, and process-relevant characteristics of representative bacterial *β*-galactosidases. Quantitative variables were standardized separately within each parameter using Z-score transformation; missing values (NR) were excluded from the corresponding mean and standard deviation. Reported ranges (e.g., 55-60 ℃) are displayed without Z-score normalization. Categorical variables (GH family) are not normalized. The color scale represents the relative numerical magnitude within a parameter and does not indicate industrial superiority. Data were compiled from the published literature and correspond to the reported experimental conditions, which differ in enzyme preparation, assay substrate, temperature, pH and analytical methodology. This figure provides a comparative overview of representative bacterial -galactosidases rather than a quantitative ranking of industrial suitability.

Quantitative variables were standardized separately within each parameter using Z-score transformation. The color scale therefore represents the relative position of each reported value within the corresponding parameter and should not be interpreted as a universal scale of industrial performance. GH-family membership and other categorical structural variables were encoded separately. Missing values were treated as unavailable data and were not assigned zero values. Because the underlying studies differ in assay substrate, matrix, temperature, pH, enzyme preparation and analytical methodology, the visualization should be interpreted as a comparative summary of reported characteristics rather than as a quantitative ranking of industrial suitability.

The integrated comparison illustrates that enzymes sharing taxonomic origin or GH-family membership do not necessarily exhibit equivalent production or process characteristics. GH2 *β*-galactosidases from lactic acid bacteria frequently combine near-neutral pH optima with catalytic properties compatible with dairy processing, whereas several thermophilic *β*-galactosidases exhibit higher temperature optima and thermal stability (Schwab et al., 2010; Kalathinathan et al., 2023). Other enzymes show favourable transgalactosylation behaviour but less extensive evidence for recombinant production or performance in real dairy matrices. These patterns should nevertheless be interpreted cautiously because the apparent grouping is influenced by both biological variation and differences in the availability and experimental comparability of published data.

Importantly, recombinant production characteristics do not consistently covary with catalytic performance. An enzyme exhibiting high catalytic activity in a purified-buffer assay may have limited soluble expression, require extensive downstream processing, or lack validation in the intended food matrix (Movahedpour et al., 2022; Liu M. et al., 2025). Conversely, an enzyme that can be secreted efficiently or deployed as an immobilized or whole-cell catalyst may offer process advantages despite having lower activity under standardized laboratory assays (Duan et al., 2023). Industrial assessment therefore requires simultaneous consideration of catalytic performance, production format, downstream-processing requirements, operational stability, and validation under application-relevant conditions.

Nevertheless, quantitative comparisons of recombinant *β*-galactosidase production at pilot or industrial scale remain scarce. Most published studies report laboratory-scale expression yields or purified-enzyme recovery, often without standardized information on volumetric productivity, recovery yield, downstream-processing cost or long-term catalyst performance. Consequently, the available production data are useful for identifying process-related advantages and limitations of different expression and catalyst formats, but they are currently insufficient for quantitative ranking of their industrial scalability.

### 5.3 Comparative industrial characteristics

Industrial evaluation of recombinant bacterial *β*-galactosidases requires matching enzyme properties to a defined process rather than identifying a universally superior catalyst. Two major applications - lactose hydrolysis in dairy matrices and transglycosylation-mediated GOS synthesis-operate under substantially different substrate concentrations, process conditions, and technological constraints. Consequently, catalytic parameters obtained under one set of conditions cannot be transferred directly to the other application (Kittibunchakul et al., 2020; Király et al., 2025).

For lactose hydrolysis in milk and whey, the relevant substrate is present within a complex dairy matrix containing proteins, minerals, salts, and other components that can modify enzyme behaviour relative to purified lactose-buffer systems. Enzymes intended for lactose-reduced or lactose-free dairy products should therefore be evaluated not only for lactose-specific catalytic efficiency but also for activity at the processing temperature and pH, stability during the required reaction period, tolerance to accumulating glucose and galactose, and experimentally demonstrated lactose conversion in the target matrix. In refrigerated processing, activity at low temperature becomes particularly important, whereas processes conducted at elevated temperatures place greater emphasis on thermostability (Benavente et al., 2015; Kittibunchakul et al., 2019).

For GOS production, product tolerance and transglycosylation capacity become more important than lactose hydrolysis alone. Recombinant *β*-galactosidase from *L. sakei* Lb790 exhibited a relatively high galactose inhibition constant (K_i_ = 180 mM) compared with *L. delbrueckii* subsp. *bulgaricus* (K_i_ = 70.7 mM), allowing prolonged catalytic activity during lactose conversion. Likewise, *β*-galactosidase from *B. bifidum* produced 107.2 g L^−1^ GOS at an initial lactose concentration of 400 g L^−1^, corresponding to a 27% GOS yield, which exceeded the yields reported for many commercial *β*-galactosidases (Iqbal et al., 2011; Nguyen et al., 2012; Füreder et al., 2020).

Thermostable enzymes represent another important industrial group because elevated reaction temperatures increase lactose solubility and reduce microbial contamination. For example, the native *β*-galactosidase from *L. helveticus* OSU-PECh-4A remained highly active between 60 and 80 °C, whereas thermophilic GH42 *β*-galactosidases from Geobacillus and related genera retain catalytic activity at approximately 70 °C, making them particularly attractive for continuous high-temperature lactose hydrolysis (Dong et al., 2011; Ruiz-Ramírez and Jiménez-Flores, 2025).

Overall, the available evidence does not identify a single *β*-galactosidase or GH family that consistently combines high catalytic efficiency, thermostability, product tolerance, recombinant productivity and demonstrated performance under industrially relevant conditions. Different enzyme groups nevertheless exhibit characteristics that may be advantageous for particular applications. GH2 *β*-galactosidases from lactic acid bacteria frequently exhibit catalytic properties compatible with near-neutral dairy-processing conditions, whereas several thermophilic GH42 enzymes show greater activity and stability at elevated temperatures. For GOS production, experimentally demonstrated transglycosylation performance under concentrated-lactose conditions is more informative than GH-family assignment alone. These trends can inform comparative evaluation, but the limited availability of standardized studies in real dairy matrices and at pilot or industrial scale currently prevents definitive ranking of enzymes for specific industrial applications.

## 6 Lactose hydrolysis and galacto-oligosaccharide synthesis

### 6.1 Catalytic mechanisms and factors influencing lactose hydrolysis

Lactose hydrolysis and transglycosylation are competing reactions catalyzed by *β*-galactosidases through a common retaining mechanism involving a covalent galactosyl-enzyme intermediate. During hydrolysis, water acts as the acceptor, resulting in the formation of glucose and galactose. In transglycosylation, a carbohydrate or another suitable nucleophile acts as the acceptor of the galactosyl residue, resulting in the formation of new glycosidic products, including galacto-oligosaccharides (GOS) (Porciuncula González et al., 2025).

The relative contribution of these pathways depends on both intrinsic enzyme properties and reaction conditions. Lactose concentration, enzyme dosage, pH, temperature, reaction time, water activity, and product accumulation influence substrate conversion and process productivity. In particular, increasing the effective concentration of suitable carbohydrate acceptors can promote transglycosylation relative to hydrolysis, whereas conditions favoring water as the competing acceptor promote hydrolytic turnover. Active-site architecture and residues involved in acceptor recognition further influence this catalytic partitioning (Jian et al., 2023; Liao et al., 2026).

### 6.2 Factors governing galacto-oligosaccharide synthesis

Efficient GOS synthesis requires reaction conditions that favor transglycosylation relative to hydrolysis. High lactose concentration is particularly important because it increases the availability of carbohydrate acceptor molecules and can thereby favor galactosyl transfer to sugars rather than water. Temperature, pH, water activity, enzyme loading, and reaction time additionally affect GOS yield and product distribution. Prolonged reaction times may also promote secondary hydrolysis of previously formed GOS, emphasizing the importance of identifying an appropriate reaction endpoint (Zhou et al., 2024; Hu et al., 2025).

The enzyme source is another major determinant of transglycosylation performance. *β*-Galactosidases belonging to different GH families, and even enzymes within the same family, may differ substantially in acceptor specificity, linkage preference, GOS yield, and degree-of-polymerization profile. Consequently, industrial GOS production requires simultaneous consideration of enzyme-specific transglycosylation properties and process conditions rather than selection based solely on hydrolytic activity (Kittibunchakul et al., 2020; Király et al., 2025).

### 6.3 Structural determinants of transglycosylation efficiency

The transglycosylation efficiency of *β*-galactosidases is strongly influenced by the architecture of the active site, including residues involved in substrate recognition, acceptor binding, and stabilization of catalytic intermediates. Changes in active-site geometry and surrounding loops can alter the accessibility and orientation of carbohydrate acceptors and consequently modify the balance between hydrolysis and transglycosylation (Hidaka et al., 2002).

Structure-guided protein engineering provides a means of modifying this balance. Recent studies demonstrate that substitutions around substrate-binding regions can alter catalytic efficiency, acceptor recognition, and GOS-forming activity, supporting the use of rational engineering for the development of *β*-galactosidases with improved transglycosylation performance (Király et al., 2025).

The relative distribution between lactose hydrolysis and transglycosylation therefore represents an important determinant of process suitability. Figure 5 summarizes the common catalytic pathway and the principal reaction and enzyme-related factors that influence partitioning between lactose hydrolysis and GOS formation.

**Figure 5:**
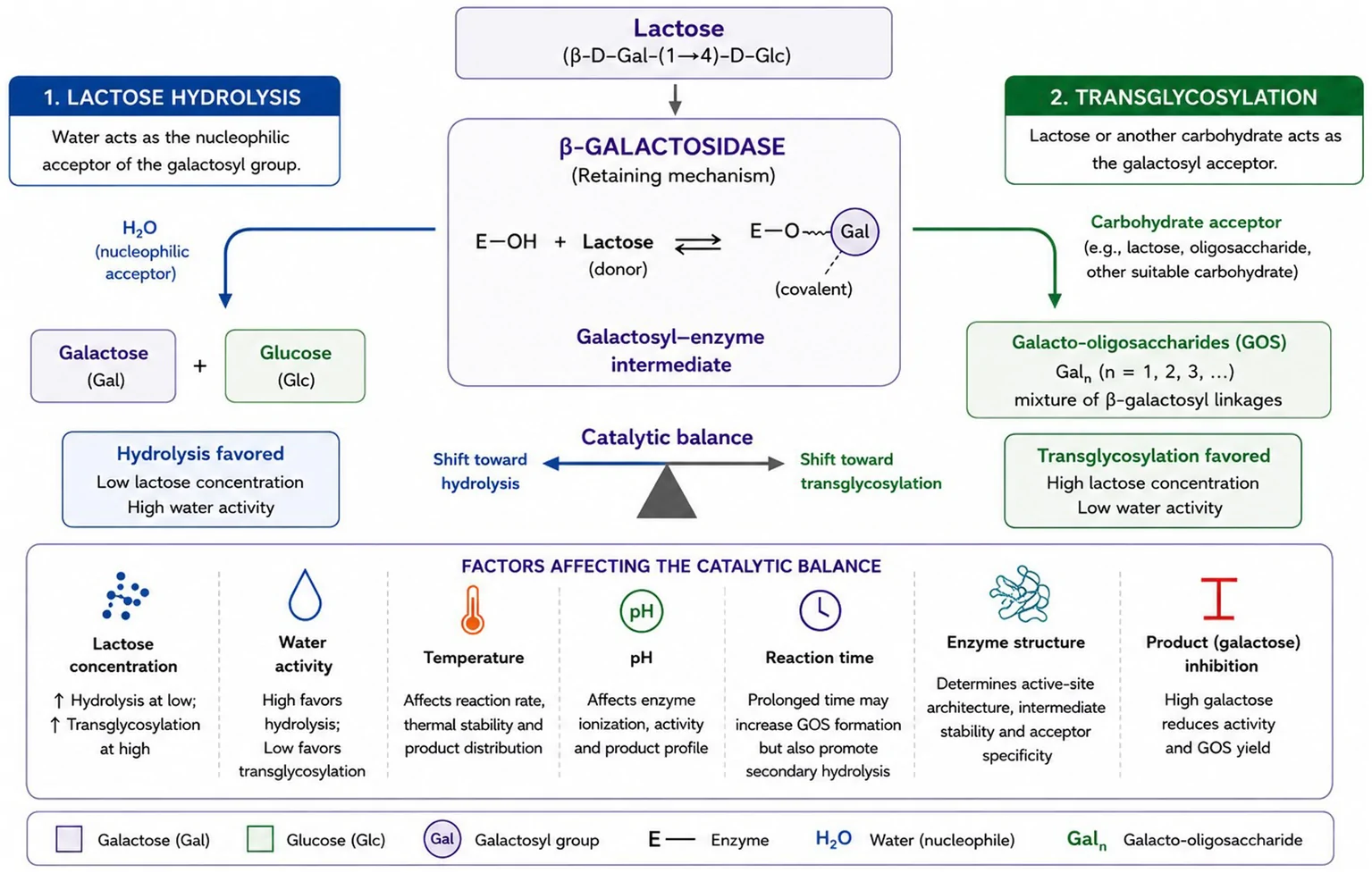
Catalytic partitioning between lactose hydrolysis and transglycosylation by *β*-galactosidase.

As illustrated in Figure 5, both lactose hydrolysis and transglycosylation originate from the same covalent galactosyl-enzyme intermediate formed during *β*-galactosidase catalysis. The reaction pathway is subsequently determined by the identity of the galactosyl acceptor. When water acts as the acceptor, the intermediate is hydrolyzed to release glucose and galactose. In contrast, when lactose or another carbohydrate molecule successfully competes with water, the galactosyl residue is transferred to the acceptor, resulting in the formation of galacto-oligosaccharides. High lactose concentration and low water activity shift the catalytic balance toward transglycosylation and increase GOS yield, whereas low substrate concentration and high water activity favor lactose hydrolysis. In addition, enzyme structure, pH, temperature, reaction time and product inhibition collectively regulate catalytic efficiency and determine the final distribution of hydrolysis and transglycosylation products.

## 7 Industrial applications and commercial *β*-galactosidases

### 7.1 Industrial applications of bacterial *β*-galactosidases

Application-relevant performance is an important consideration when evaluating the technological potential of recombinant bacterial *β*-galactosidases. Their industrial relevance extends beyond efficient lactose hydrolysis, contributing to more sustainable food manufacturing through reduced processing severity, improved product quality and the valorization of dairy side streams, thereby decreasing reliance on intensive chemical treatments (Kostanova et al., 2025). *β*-Galactosidases are widely employed in the production of lactose-free dairy products, while recombinant bacterial enzymes are increasingly investigated for lactose conversion, whey bioconversion and galacto-oligosaccharide (GOS) synthesis. In addition, GOS-producing *β*-galactosidases have become important biocatalysts for functional foods and infant formula because of the well-established prebiotic properties of GOS (Buchanan et al., 2023; Kalathinathan et al., 2023).

Whey and whey permeate should not be considered simply as low-value waste streams because modern dairy processing already valorizes whey proteins, lactose, permeate powders, and other fractions. In this context, *β*-galactosidase treatment represents an additional strategy for converting lactose-rich dairy streams into lactose-reduced products or value-added oligosaccharides. Feedstock composition and lactose concentration are therefore important process variables. Direct treatment of milk and relatively dilute dairy streams is predominantly associated with lactose hydrolysis, whereas efficient GOS manufacture generally requires concentrated lactose feeds. Published GOS processes employ lactose concentrations ranging from approximately 200 to >500 g L^−1^ depending on the enzyme and process configuration, clearly distinguishing GOS manufacture from conventional lactose hydrolysis in milk (Palai et al., 2012; Teng et al., 2024).

Catalyst format constitutes an additional industrial decision point. Soluble enzymes provide straightforward batch processing, whereas immobilization or enzyme-retention systems can facilitate catalyst recovery and repeated use. Immobilized recombinant *Lactobacillus bulgaricus β*-galactosidase, for example, produced 49% GOS from 400 g L^−1^ lactose and retained more than 85% activity after 20 reaction cycles, demonstrating the potential advantages of catalyst reuse (Lu et al., 2012).

Consequently, comparative evaluation of *β*-galactosidases for industrial applications should consider the intended feedstock, lactose concentration, catalyst configuration, operational stability, product inhibition, enzyme recovery or reuse and downstream-processing requirements, rather than relying exclusively on catalytic activity measured under laboratory conditions (Lu et al., 2012; Teng et al., 2024).

### 7.2 Commercial *β*-galactosidase preparations

Commercial *β*-galactosidases originate from diverse microbial sources and differ substantially in their operating conditions and application profiles. Neutral yeast *β*-galactosidases from *Kluyveromyces* spp. have long been used for lactose conversion under near-neutral conditions, whereas fungal *β*-galactosidases from *Aspergillus* spp. provide complementary activity under more acidic conditions. Commercial bacterial *β*-galactosidases, including preparations originating from *B. bifidum* and *B. circulans*, further expand the available range of hydrolytic and transglycosylation properties (Palai et al., 2012; Füreder et al., 2020; Teng et al., 2024).

Importantly, commercial preparations developed for lactose hydrolysis cannot automatically be assumed to perform equally well in GOS manufacture. Their transglycosylation performance depends strongly on enzyme origin, lactose concentration, enzyme loading, temperature, pH, reaction time, and reactor configuration. Comparative studies of commercial *β*-galactosidases have consequently reported substantial differences in GOS productivity, demonstrating the need to evaluate commercial preparations under application-specific conditions rather than extrapolating GOS potential from hydrolytic activity alone (Füreder et al., 2020; Teng et al., 2024).

A particularly relevant example is Saphera, a commercial *B. bifidum β*-galactosidase. At relatively low lactose concentrations its activity is predominantly hydrolytic, whereas increasing the initial lactose concentration to 400 g L^−1^ resulted in the formation of 107.2 g L^−1^ GOS, corresponding to a yield of approximately 27%. Maximum GOS formation occurred at approximately 90% lactose conversion, illustrating that the balance between hydrolysis and transglycosylation is strongly dependent on reaction conditions even for the same commercial enzyme (Füreder et al., 2020).

Commercial recombinant bacterial *β*-galactosidases also provide direct evidence that recombinant enzyme technology can be translated into food-processing applications. Saphera 2,600 L and NOLA Fit 5,500 are commercially available preparations containing recombinant *B. bifidum β*-galactosidase produced using *B. licheniformis*. Both preparations have been evaluated directly in pasteurized milk as free and chitosan-immobilized enzymes. Immobilization improved their operational stability, and the enzyme-coated beads could be reused for at least 15 cycles while maintaining residual lactose concentrations below 2 mg mL^−1^ after each cycle. GOS accounted for up to 9% of total sugars in the treated milk (Bodnár et al., 2024).

Commercial fungal *β*-galactosidases provide a complementary technological option for acidic processing environments. In addition, regulatory assessment provides direct evidence that recombinant fungal production systems can be considered for food applications. EFSA evaluated *β*-galactosidase produced with genetically modified *Aspergillus niger* strain TOL for three manufacturing processes: production of lactose-reduced dairy products, fermented dairy products, and oligosaccharides. For GOS production, the assessed raw material was concentrated liquid acid whey containing 15% (w/w) lactose. The enzyme exhibited an activity optimum of approximately 55 °C at pH 4.5 and a pH optimum of approximately 4.0–4.5 (Zorn et al., 2025).

A comparative overview of representative commercial *β*-galactosidase preparations is presented in Table 6.

**Table 6:** Representative commercial *β*-galactosidase preparations and industrially relevant catalysts for lactose hydrolysis and GOS production.

Enzyme/preparation	Manufacturer	Enzyme source	Catalyst/production format	Application	Quantitative application evidence	Reference
Lactozym® Pure 6,500 L	Novozymes	*Kluyveromyces lactis*	Commercial soluble *β*-galactosidase	Lactose conversion; GOS synthesis	Evaluated in concentrated-lactose free-enzyme membrane-reactor systems	Teng et al. (2024)
Saphera® 2,600 L	Novozymes	*Bifidobacterium bifidum*	Recombinant *β*-galactosidase produced using *Bacillus licheniformis*	Dairy lactose hydrolysis; GOS synthesis	Milk: immobilized enzyme reusable for ≥15 cycles, residual lactose <2 mg mL^−1^ and GOS ≤ 9% of total sugars; concentrated lactose: 400 g L^−1^ → 107.2 g L^−1^ GOS (~27% yield)	Bodnár et al. (2024)
Saphera®	Novonesis	*Bifidobacterium bifidum*	Commercial soluble *β*-galactosidase	GOS synthesis	400 g L^−1^ lactose → 107.2 g L^−1^ GOS (~27% yield); maximum GOS at ~90% lactose conversion	Füreder et al. (2020)
NOLA™ Fit 5,500	Chr. Hansen (Novonesis)	*Bifidobacterium bifidum*	Recombinant *β*-galactosidase produced using *Bacillus licheniformis*	Dairy lactose hydrolysis; GOS synthesis	In milk, immobilized enzyme reusable for ≥15 cycles; residual lactose <2 mg mL^−1^; GOS ≤ 9% total sugars	Bodnár et al. (2024)
Lactase F	Amano	*Aspergillus oryzae*	Commercial fungal *β*-galactosidase	Acidic lactose conversion; GOS synthesis	Evaluated at concentrated lactose levels in membrane-reactor systems; maximum GOS yield reported up to 35.6 wt%	Teng et al. (2024)
Enzeco® Fungal Lactase	Enzyme Development Corporation	*Aspergillus oryzae*	Commercial fungal *β*-galactosidase	GOS synthesis	Approximately 30 wt% GOS reported from concentrated lactose under membrane-reactor conditions	Teng et al. (2024)
Gamma-lactase A50P	Gamma Chemie GmbH	*Aspergillus oryzae*	Commercial fungal *β*-galactosidase	GOS synthesis	Approximately 15–18 wt% GOS depending on membrane-reactor configuration	Teng et al. (2024)
Biolacta FN5	Daiwa Kasei K. K.	*Bacillus circulans*	Commercial bacterial *β*-galactosidase	GOS synthesis	525 g L^−1^ lactose → 39% GOS at 60% lactose conversion	Palai et al. (2012)
*β*-Galactosidase from strain TOL	DSM Food Specialties B. V.	GM *Aspergillus niger* TOL	Recombinant food-enzyme production strain	Lactose-reduced dairy; fermented dairy; GOS production	GOS application assessed using concentrated acid whey containing 15% (w/w) lactose; optimum ~55 °C at pH 4.5	Zorn et al. (2025)
*K. marxianus* NCIM 3551 *β*-galactosidase^1^	Experimental benchmark	*Kluyveromyces marxianus*	Permeabilized whole-cell catalyst	GOS synthesis	200 g L^−1^ lactose → 36% GOS; 80% lactose conversion; 24 g L^−1^ h^−1^ productivity	Srivastava et al. (2015)

Table 6 highlights the substantial diversity of commercial *β*-galactosidase preparations with respect to enzyme source, catalyst format, and application profile. Importantly, preparations effective for lactose hydrolysis do not necessarily provide the highest GOS productivity, which depends strongly on substrate concentration, enzyme origin, and process configuration. Commercial bacterial enzymes such as Saphera and Biolacta FN5 demonstrate the industrial relevance of bacterial *β*-galactosidases, whereas yeast- and fungal-derived preparations extend the range of operating conditions available for dairy processing and GOS synthesis (Palai et al., 2012; Bodnár et al., 2024; Teng et al., 2024). These differences emphasize the importance of evaluating commercial enzymes under application-specific conditions rather than comparing them on the basis of a single catalytic characteristic.

### 7.3 Current technological limitations of commercial *β*-galactosidases

Despite the successful industrial implementation of *β*-galactosidases, their technological limitations are strongly application-dependent. An enzyme that performs efficiently in lactose hydrolysis cannot necessarily be expected to provide high GOS productivity because hydrolysis and transglycosylation impose different requirements on substrate concentration and catalytic partitioning. Depending on the enzyme and process, product inhibition, insufficient operational stability, enzyme dosage, catalyst recovery, matrix effects, and process economics may constrain industrial performance (Füreder et al., 2020; Teng et al., 2024).

Although these preparations are highly effective for lactose hydrolysis, relatively few commercial enzymes have been specifically optimized for high-yield GOS production or combine high thermostability with reduced product inhibition. These limitations continue to drive the development of improved recombinant bacterial *β*-galactosidases for industrial biotechnology.

This distinction is particularly important for GOS manufacture. Commercial preparations exhibit markedly different GOS yields under concentrated-lactose conditions. For example, Saphera produced approximately 27% GOS from 400 g L^−1^ lactose, whereas commercial *B. circulans* Biolacta FN5 reached 39% GOS from 525 g L^−1^ lactose under the conditions reported by Palai et al. (2012). These values should not be interpreted as a direct ranking because enzyme dosage, substrate concentration, reaction conditions, and analytical procedures differ among studies. Rather, they demonstrate that transglycosylation performance must be evaluated independently from conventional lactose-hydrolysis activity (Palai et al., 2012; Füreder et al., 2020).

Catalyst deployment represents another major determinant of industrial feasibility. His-tag purification followed by immobilized metal affinity chromatography (IMAC), fast protein liquid chromatography (FPLC), or size-exclusion chromatography is highly useful for biochemical characterization of recombinant proteins but does not necessarily represent the preferred configuration for large-scale food-enzyme manufacture. More scalable strategies include concentrated soluble preparations, immobilized catalysts, enzyme-retention membrane systems, and whole-cell or permeabilized-cell catalysts. Immobilization can facilitate catalyst recovery and repeated use, as demonstrated for recombinant *L. bulgaricus β*-galactosidase immobilized on cellulose and for commercial Saphera and NOLA preparations immobilized on chitosan beads (Lu et al., 2012; Bodnár et al., 2024). Free-enzyme membrane reactors provide another strategy for enzyme retention and process intensification in GOS manufacture (Teng et al., 2024).

Whole-cell catalysis can further reduce enzyme-isolation and purification requirements, although transport of lactose and reaction products across the cellular envelope may limit apparent catalytic performance. Permeabilized *Kluyveromyces marxianus* NCIM 3551 cells, for example, produced 36% GOS from 200 g L^−1^ lactose with 80% lactose conversion and a productivity of 24 g L^−1^ h^-1^. These results illustrate the technological potential of alternative catalyst formats and reinforce the need to evaluate catalyst deployment together with intrinsic enzyme properties (Srivastava et al., 2015).

Food-industry implementation additionally requires consideration of the production organism, manufacturing process, and regulatory status of recombinant enzyme preparations. Recombinant production itself does not preclude food use; rather, safety assessment considers the production strain, introduced genetic material, manufacturing process, enzyme preparation, intended use, and consumer exposure. In the European Union, food enzymes are regulated under Regulation (EC) No 1332/2008 (European Parliament and Council, 2008; European Commission, n.d.). EFSA subsequently evaluated *β*-galactosidase produced with genetically modified *A. niger* strain TOL and concluded that the enzyme did not raise safety concerns under the revised intended conditions of use, including lactose-reduced dairy products, fermented dairy products, and oligosaccharide production (Zorn et al., 2025).

Commercial Saphera and NOLA preparations provide an additional example relevant to recombinant bacterial *β*-galactosidases. Both contain recombinant *B. bifidum β*-galactosidase produced using *B. licheniformis* and have been evaluated directly in pasteurized milk in both free and immobilized forms. Their performance in a real dairy matrix illustrates the importance of validating recombinant enzymes under application-relevant conditions rather than relying exclusively on assays conducted in buffers or with chromogenic substrates (Bodnár et al., 2024).

Future *β*-galactosidase development should therefore prioritize application-specific combinations of properties rather than maximization of a single catalytic parameter. For lactose hydrolysis, desirable characteristics include high matrix-specific conversion, appropriate pH and temperature compatibility, operational stability, low enzyme dosage, and cost-effective production. For GOS manufacture, priorities shift toward high transglycosylation efficiency at concentrated lactose levels, reduced secondary GOS hydrolysis, favorable product profiles, operational stability, and efficient catalyst recovery or reuse. Structure-guided and AI-assisted protein engineering may further facilitate the development of enzymes combining these characteristics, but industrial relevance ultimately requires validation using realistic feedstocks, food-compatible production systems, and scalable catalyst configurations (Király et al., 2025; Liao et al., 2026).

Figure 6 summarizes the current applications, application-dependent technological limitations, and future industrial requirements of commercial *β*-galactosidases.

**Figure 6:**
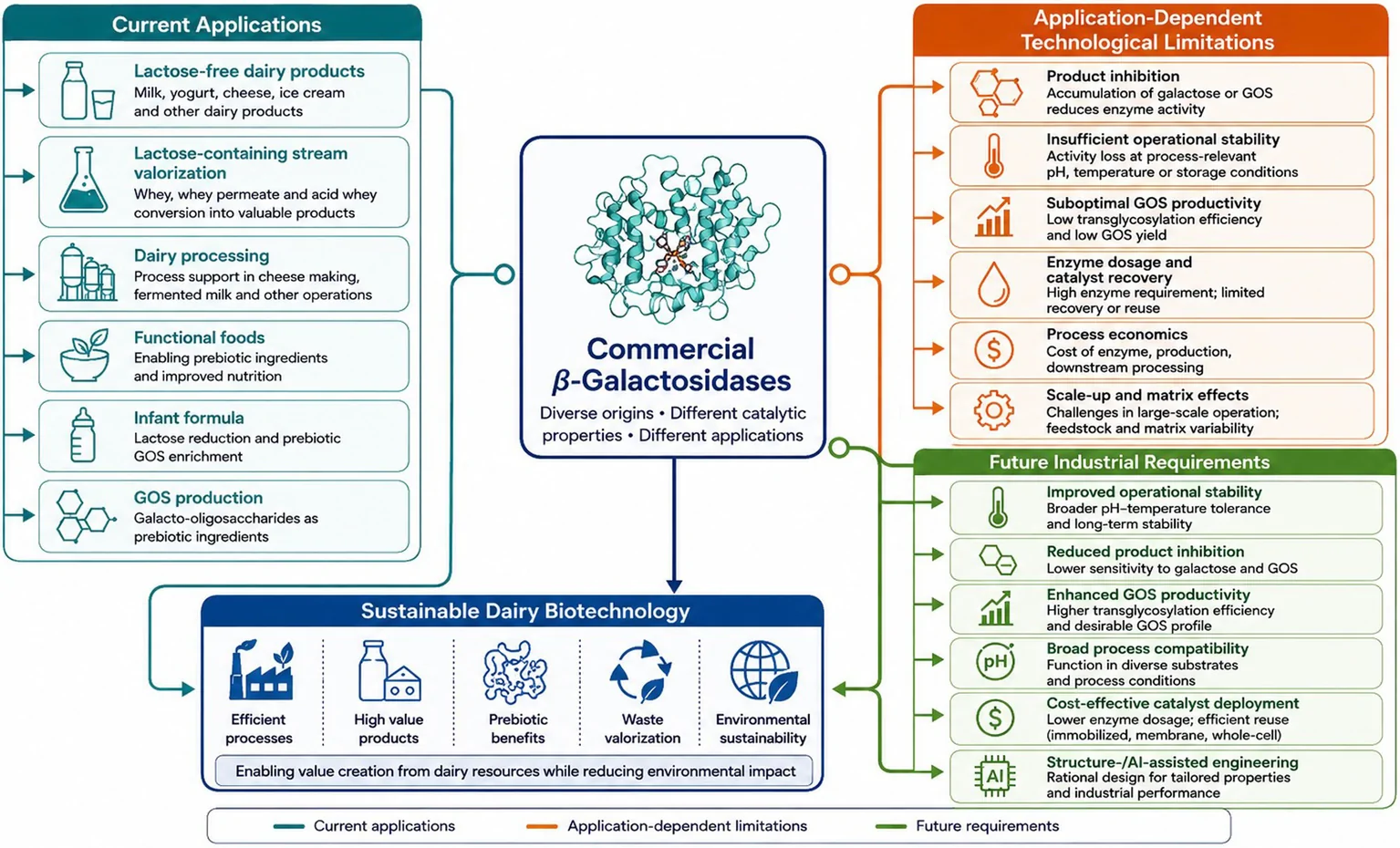
Commercial *β*-galactosidases: current applications, application-dependent technological limitations and future industrial requirements.

Figure 6 summarizes the current industrial applications of commercial *β*-galactosidases, the major technological limitations affecting their performance and the key characteristics required for next-generation recombinant enzymes. Current applications include lactose hydrolysis in dairy products, valorization of lactose-containing dairy streams, and transglycosylation-mediated GOS production. Depending on the enzyme and process, technological constraints may include product inhibition, insufficient operational stability, suboptimal GOS productivity, enzyme dosage and catalyst recovery, process economics, and scale-up or matrix effects. Future development should integrate improved operational stability, reduced product inhibition, enhanced GOS productivity, broad process compatibility, cost-effective catalyst deployment, and structure- or AI-assisted enzyme engineering. The application-dependent limitations of commercial *β*-galactosidases demonstrate that industrial enzyme selection requires integration of catalytic properties with feedstock characteristics, catalyst format, production strategy, and process economics. Taken together, these application-dependent limitations demonstrate that the technological evaluation of recombinant bacterial *β*-galactosidases requires consideration of catalytic properties together with feedstock characteristics, catalyst format, production strategy and process economics. The following section therefore compares the principal application-related criteria reported for recombinant bacterial *β*-galactosidases in the context of lactose hydrolysis, GOS production and other relevant processing conditions, while recognizing the limitations imposed by heterogeneous and predominantly laboratory-scale evidence.

## 8 Application-oriented comparison of recombinant bacterial *β*-galactosidases

### 8.1 Application-related considerations for recombinant *β*-galactosidases

The comparative evaluation of recombinant bacterial *β*-galactosidases should begin with the intended application rather than with glycoside hydrolase (GH) family or catalytic activity measured using chromogenic substrates. Lactose hydrolysis in milk or whey, GOS synthesis from concentrated lactose, and processing at elevated temperature impose substantially different requirements on enzyme performance. Consequently, candidate enzymes should be evaluated using application-relevant substrates and process conditions whenever such data are available.

For lactose hydrolysis, the principal criteria include efficient conversion of lactose in the target dairy matrix, activity and stability at the required processing pH and temperature, tolerance to glucose and galactose, and sufficient operational lifetime. Lactose-specific kinetic parameters can support preliminary comparison, but they should not be used as stand-alone predictors of industrial performance because reported values are strongly influenced by assay conditions and do not account for matrix effects, enzyme stability, or catalyst configuration (Kittibunchakul et al., 2019).

For GOS manufacture, the evaluation criteria shift toward transglycosylation performance under concentrated-lactose conditions. Relevant parameters include maximum GOS yield, lactose conversion at maximum GOS formation, product profile, resistance to product inhibition, operational stability, and the extent of secondary GOS hydrolysis. Enzyme source and GH family can guide comparative evaluation but should not substitute for experimentally measured transglycosylation performance (Kittibunchakul et al., 2020).

### 8.2 Comparative performance criteria according to application

To facilitate comparative evaluation, qualitative descriptors such as «high catalytic efficiency», «high GOS yield» and «high thermostability» can be considered in relation to measurable performance parameters. Because published *β*-galactosidase studies employ heterogeneous assay conditions, the proposed values should be interpreted as comparative reference points rather than universal biochemical or industrial thresholds.

For lactose hydrolysis, priority should be given to enzymes demonstrating substantial lactose conversion directly in milk, whey, or another target dairy matrix under process-relevant conditions. Lactose-specific *K*_m_, *k*_cat_, and *k*_cat_/*K*_m_ values should be considered together with operational stability and product inhibition rather than independently. For GOS synthesis, a practical benchmark of ≥30% GOS yield relative to initial or total carbohydrate, according to the definition used in the original study, under concentrated-lactose conditions can be used to identify promising candidates for further process optimization. Several recombinant and commercial *β*-galactosidases reach approximately 30–40% or higher under appropriate conditions, although direct comparison requires consideration of substrate concentration and yield definition (Iqbal et al., 2011; Füreder et al., 2020; Yañez-Ñeco et al., 2021).

For high-temperature processing, operational activity and stability at ≥60 °C provide a useful screening criterion rather than GH-family assignment alone. Thermostable GH42 *β*-galactosidases such as BgaB from *G. stearothermophilus* illustrate this phenotype, but substantial differences in catalytic efficiency and thermostability occur even within GH42 enzymes. Therefore, thermostability should be experimentally demonstrated rather than inferred solely from phylogeny or GH classification.

### 8.3 Integrated comparative assessment

The potential industrial application of recombinant bacterial *β*-galactosidases depends on multiple interconnected biochemical, production-related and process-related characteristics. Since no single recombinant *β*-galactosidase simultaneously possesses all desirable catalytic and technological properties, comparative assessment should consider the intended application and the conditions under which the enzyme is expected to operate. Catalytic efficiency, thermostability, transglycosylation capacity, product inhibition, recombinant protein yield, purification strategy, food-production compatibility and process economics should therefore be considered together (Movahedpour et al., 2022; Kalathinathan et al., 2023; Király et al., 2025).

Table 7 summarizes the principal application-relevant parameters and comparative reference points reported for major technological applications of recombinant bacterial *β*-galactosidases.

**Table 7:** Comparative criteria for evaluating recombinant bacterial *β*-galactosidases according to application.

Industrial scenario	Primary evaluation parameter	Comparative reference point	Additional selection criteria	Representative evidence
Lactose hydrolysis in milk/whey	Matrix-specific lactose conversion	Demonstrated substantial lactose conversion directly in the target dairy matrix under process-relevant conditions	Appropriate pH/T range; operational stability; glucose/galactose tolerance; enzyme dosage	Kittibunchakul et al. (2019), Yañez-Ñeco et al. (2021)
GOS production	Transglycosylation performance	≥30% GOS yield under concentrated-lactose conditions as a literature-informed screening reference point*	Lactose conversion; product profile; limited secondary hydrolysis; product tolerance; operational stability	Iqbal et al. (2010), Yañez-Ñeco et al. (2021)
High-temperature dairy processing	Thermal activity and operational stability	Catalytic activity and experimentally demonstrated stability at ≥60 °C*	Half-life at process temperature; pH stability; product tolerance	Dong et al. (2011), Aulitto et al. (2021)
Food-grade applications	Production-system suitability	Food-compatible host/process and regulatory acceptability for intended use	Absence of undesirable selection markers; secretion where advantageous; scalable recovery	Kittibunchakul et al. (2019), Zhou et al. (2024)
Large-scale industrial production	Recombinant productivity and downstream feasibility	High soluble or secreted production demonstrated at preparative scale; no universal numerical cut-off proposed	Catalyst recovery/reuse; avoidance of chromatography-intensive purification where possible; production cost	Duan et al. (2023), Zhou et al. (2024)

The numerical values presented are comparative reference points derived from published studies rather than universally validated industrial thresholds. Published studies differ in substrate concentration, analytical methods, enzyme dosage, reaction time, and definitions of GOS yield. Consequently, quantitative values should be interpreted together with the experimental conditions reported in the original studies. GH-family assignment is considered a supporting characteristic rather than an independent selection criterion.

Table 7 demonstrates that application-related performance cannot be evaluated on the basis of a single enzyme property or GH-family assignment. For lactose hydrolysis, direct performance in the target dairy matrix provides stronger application evidence than catalytic activity measured exclusively with chromogenic substrates. For GOS production, transglycosylation performance under concentrated-lactose conditions becomes the primary criterion, whereas high-temperature processes require experimentally demonstrated thermal stability. Recombinant productivity, catalyst configuration, downstream processing, and food-production compatibility subsequently determine whether a catalytically promising enzyme can be translated into an industrially feasible biocatalyst.

However, heterogeneity in experimental conditions, limited testing in real dairy matrices and the scarcity of pilot- and industrial-scale comparative studies currently preclude definitive quantitative ranking of recombinant *β*-galactosidases for industrial applications.

## 9 Future perspectives

### 9.1 Rational protein engineering of *β*-galactosidases

Rational protein engineering provides a targeted strategy for improving *β*-galactosidase properties that currently limit industrial performance, including insufficient thermostability, product inhibition, suboptimal catalytic efficiency, and an unfavorable balance between hydrolysis and transglycosylation. Rather than modifying enzymes solely on the basis of global sequence similarity, contemporary approaches increasingly use structural information to identify residues involved in substrate recognition, active-site geometry, conformational flexibility, oligomeric interactions, and thermal stability. Site-directed mutagenesis, loop engineering, modification of flexible regions, and stabilization of intersubunit interactions can therefore be used to tailor *β*-galactosidases toward specific process requirements.

Recent experimental evidence demonstrates the potential of this approach. Structure-guided engineering of *Kluyveromyces lactis β*-galactosidase generated the L764T and L764T/V842G variants with 2.9- and 4.8-fold increases in catalytic efficiency (*k*_cat_/*K*_m_), respectively. The double mutant also increased GOS yield from 35.2% for the wild-type enzyme to 47.9%. Molecular-dynamics analysis associated these improvements with altered substrate binding, increased rigidity of specific regions, and strengthened intersubunit interactions (Liao et al., 2026).

Although this recent example involves a yeast-derived *β*-galactosidase, it provides a relevant proof of principle for structure-guided strategies applicable to recombinant bacterial enzymes. Future engineering of bacterial *β*-galactosidases should therefore connect structural modifications directly with application-relevant endpoints, including lactose conversion in dairy matrices, GOS yield and product profile, operational half-life, and resistance to glucose and galactose inhibition. This is particularly important because improvements detected with chromogenic substrates do not necessarily predict performance with lactose under industrially relevant conditions (Kittibunchakul et al., 2019; Király et al., 2025)

### 9.2 Directed evolution and high-throughput screening

Directed evolution represents a complementary protein-engineering strategy when the structural determinants of a desired phenotype are incomplete or difficult to predict. In contrast to purely rational modification, iterative sequence diversification and screening can identify beneficial mutations without requiring complete prior knowledge of structure–function relationships. Rational design and directed evolution should therefore be considered parallel and potentially complementary approaches within the broader field of protein engineering rather than sequential hierarchical levels.

For *β*-galactosidases, an important limitation is the choice of screening endpoint. Screening based exclusively on ONPG or other chromogenic substrates may identify variants with increased apparent hydrolytic activity without necessarily improving lactose conversion or transglycosylation under application-relevant conditions. Future screening pipelines should therefore incorporate lactose as the substrate and, where feasible, secondary assays quantifying lactose conversion, GOS yield and composition, thermal stability, and glucose or galactose inhibition. Such application-oriented screening would improve the probability that variants selected at laboratory scale retain their advantages under industrial processing conditions (Kittibunchakul et al., 2019; Király et al., 2025).

The integration of focused saturation mutagenesis, high-throughput screening, and computational prioritization offers a route to reducing the experimental search space. Rather than screening increasingly large libraries without application-specific criteria, future workflows should use experimentally generated sequence–function relationships to guide subsequent rounds of diversification. This iterative strategy provides a conceptual bridge between conventional directed evolution and data-driven enzyme design and is particularly relevant when several properties-including catalytic activity, thermostability, transglycosylation, and product tolerance-must be optimized simultaneously (Király et al., 2025).

### 9.3 AI-assisted and computational enzyme engineering

Artificial intelligence and computational modelling are increasingly expanding the design space available for enzyme engineering. Structure prediction, molecular-dynamics simulations, sequence analysis, and machine-learning approaches can assist in identifying flexible regions, substrate-interacting residues, stability determinants, and potentially beneficial substitutions before experimental testing. These approaches are particularly valuable for *β*-galactosidases lacking experimentally determined structures and can reduce the number of variants requiring experimental characterization (Király et al., 2025; Liao et al., 2026).

However, structural prediction should not be equated with experimental demonstration of enzyme function. Computational models are most informative when integrated with biochemical characterization and experimentally determined sequence–function relationships. The recent engineering of *K. lactis β*-galactosidase illustrates such an integrated approach: sequence analysis and structural modelling were combined with mutagenesis, kinetic measurements, GOS-production experiments, and molecular-dynamics simulations to identify variants with improved catalytic efficiency and transglycosylation performance (Liao et al., 2026).

For recombinant bacterial *β*-galactosidases, future computational pipelines should move toward multi-objective optimization integrating structural information with experimentally measured K*_m_, k_cat_, k_cat_/K_m_*, GOS yield and product profile, thermal half-life, product inhibition, soluble recombinant expression, and performance in application-relevant matrices. Such an approach is preferable to optimizing a single property because catalytic efficiency, stability, transglycosylation, substrate specificity, and recombinant productivity may impose competing constraints on industrial enzyme performance (Király et al., 2025).

### 9.4 Future industrial implementation and sustainable dairy biorefineries

The major challenge for next-generation recombinant bacterial *β*-galactosidases is the transition from improved laboratory phenotypes to economically and technologically viable industrial biocatalysts. Future development should therefore progress through staged validation rather than terminating after biochemical characterization of purified recombinant proteins. Following enzyme discovery and engineering, promising candidates should be evaluated using realistic feedstocks, including milk, whey, whey permeate, or concentrated lactose streams according to the intended application. Catalyst format, operational stability, enzyme recovery or reuse, downstream-processing requirements, and recombinant productivity should subsequently be evaluated before pilot-scale implementation (Kittibunchakul et al., 2019; Király et al., 2025).

For lactose hydrolysis, future development should emphasize matrix-specific lactose conversion, compatibility with dairy-processing conditions, operational stability, and economical enzyme production. For GOS manufacture, priorities shift toward high transglycosylation efficiency at concentrated lactose levels, favorable product profiles, reduced secondary GOS hydrolysis, catalyst recovery or reuse, and integration with product-separation technologies. Enzyme-retention membrane reactors represent one possible process-intensification strategy because they enable enzyme recovery and reuse while potentially avoiding repeated enzyme inactivation and removal; however, stable long-term product quality and scale-up remain important considerations (Teng et al., 2024).

Commercial recombinant *B. bifidum β*-galactosidases evaluated directly in milk further demonstrate the importance of connecting recombinant production with realistic food-matrix validation (Bodnár et al., 2024).

Ultimately, recombinant bacterial *β*-galactosidases could contribute to integrated dairy biorefineries in which lactose-containing streams are directed toward lactose-reduced products, functional GOS ingredients, or other value-added products according to feedstock composition and process requirements. Achieving this transition will require coordinated development of enzyme engineering, food-compatible recombinant production, catalyst deployment, process intensification, and quantitative assessment of economic and environmental performance (Teng et al., 2024; Király et al., 2025).

To translate these priorities into a development pathway, Figure 7 proposes a staged roadmap from enzyme discovery and engineering to application-oriented validation, scale-up and longer-term integration into sustainable dairy biorefineries. Progression between stages should be based on measurable performance criteria, including improved catalytic or transglycosylation performance relative to the parental enzyme, demonstrated operational stability under target process conditions, validation in application-relevant feedstocks, scalable recombinant production, and pilot-scale assessment of catalyst productivity and process economics.

**Figure 7:**
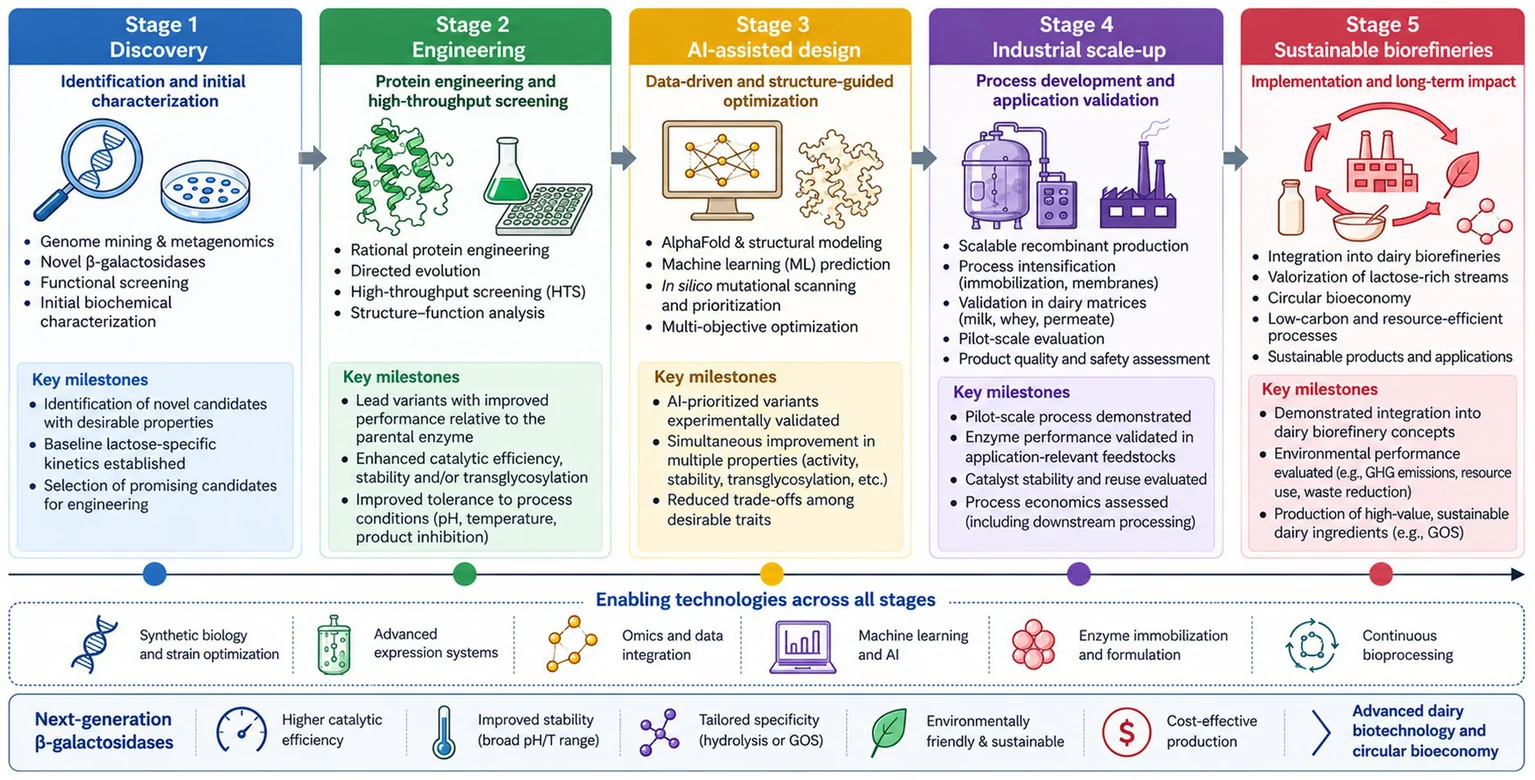
Proposed roadmap for the development and industrial translation of next-generation recombinant bacterial *β*-galactosidases.

The roadmap presents prospective stages encompassing enzyme discovery, protein engineering and high-throughput screening, AI-assisted multi-objective design, pilot-to-industrial scale-up, and longer-term integration into sustainable dairy biorefineries. Progression between stages is linked to experimentally assessable milestones, including improved catalytic or transglycosylation performance, operational stability, recombinant production, validation in application-relevant matrices, catalyst recovery or reuse, and process-economic and sustainability assessment. The roadmap represents a prospective research and development pathway rather than a prediction of fixed technological timelines.

## 10 Conclusion

Bacterial *β*-galactosidases represent important biocatalysts for lactose hydrolysis and transglycosylation-mediated galacto-oligosaccharide (GOS) synthesis, with applications in lactose-reduced dairy products, functional ingredients and the valorization of lactose-containing dairy streams. Advances in genome mining, recombinant expression, protein engineering and computational approaches have substantially expanded the range of enzymes available for these applications.

This review demonstrates that the potential industrial application of recombinant bacterial *β*-galactosidases cannot be inferred from catalytic activity alone. Comparative evaluation requires consideration of lactose-specific kinetics together with operational stability, product inhibition, transglycosylation capacity, recombinant productivity, catalyst format, food-production compatibility and performance in application-relevant substrates. The relative importance of these characteristics depends on the intended process: lactose hydrolysis requires efficient conversion and stability in dairy matrices, whereas GOS manufacture requires strong transglycosylation performance under concentrated-lactose conditions and control of secondary product hydrolysis. Accordingly, kinetic parameters obtained in buffered systems should be complemented by validation in real food matrices, while recombinant production strategies should be evaluated together with secretion capability, scalable downstream processing, regulatory suitability and process economics.

By integrating structural, catalytic, recombinant-production and process-level evidence, this review provides a comparative perspective on the factors relevant to the technological evaluation of recombinant bacterial *β*-galactosidases. The comparative kinetic and GOS datasets, functional heatmap and application-oriented criteria highlight both potentially relevant enzyme characteristics and important gaps in the current literature. In particular, standardized kinetic datasets, direct validation in milk and whey, comparable GOS-production studies and scale-relevant information on recombinant productivity and catalyst deployment remain limited. Future studies should therefore integrate lactose-specific kinetic characterization with real-matrix validation, scalable recombinant production and food-compatible downstream processing rather than evaluating these parameters independently. Consequently, the available evidence is useful for comparative assessment but does not yet support definitive quantitative ranking of recombinant *β*-galactosidases for industrial applications.
