# Supplementary material for: Recombinant bacterial β-galactosidases for lactose bioconversion and galacto-oligosaccharide production: a comparative overview
Source: Front Microbiol. 2026 Oct 12;17:1943869. doi: 10.3389/fmicb.2026.1943869 (PMC13697599; doi:10.3389/fmicb.2026.1943869)
Supplement: Supplementary file 1 [file Supplementary_file_1.docx]

**Supplementary Figure S1**

Phylogenetic relationships among representative GH2 LacZ β-galactosidases based on full-length amino-acid sequences. Bootstrap support values are shown at internal nodes, and branch lengths represent amino-acid substitutions per site. Taxon colours indicate bacterial genera. The tree illustrates sequence relationships among the selected GH2 LacZ proteins and should not be interpreted as bacterial species phylogeny or as a direct predictor of industrial performance.
